# Discovery of actinators, actin-derived bioactive peptides that modulate cytoskeleton and actin-related cellular activities

**DOI:** 10.1126/sciadv.aeb5548

**Published:** 2026-04-17

**Authors:** Fei Yi, Jia Guo, Thuy T. Vo, Brian Hetrick, Amrita Haikerwal, Zheng Zhou, Leanna Sealey, Sijia He, Yang Han, Linda Chilin, Mark Spear, Dongyang Yu, Yuriy Kim, Fatah Kashanchi, Tongqing Zhou, Xuehua Xu, Christopher Lockhart, Yuntao Wu

**Affiliations:** ^1^ Center for Infectious Disease Research, George Mason University, Manassas, VA 20110, USA.; ^2^ Vaccine Research Center, National Institute of Allergy and Infectious Diseases, National Institutes of Health, Bethesda, MD 20892, USA.; ^3^ Chemotaxis Signal Section, Laboratory of Immunogenetics, National Institute of Allergy and Infectious Diseases, National Institutes of Health, Rockville, MD 20852, USA.; ^4^ School of Systems Biology, George Mason University, Manassas, VA 20110, USA.

## Abstract

The cortical actin cytoskeleton serves as a structural support and force-generating machinery in cells, but the actin meshwork can also act as a barrier, hindering the delivery of extracellular substances. To address the issue of the cortical actin barrier, we engineered synthetic libraries of human β-actin–derived peptides to promote actin dynamics. Here, we report the discovery of six major peptides, collectively termed actinators, that are biologically active in selective modulation of actin-related cellular activities. Using retro- and lentiviral vector transduction of CAR T cells as a model, we demonstrate that an actinator markedly enhances viral vector transduction 20- to 30-fold. An individual actinator can also selectively modulate cellular functions such as regulating surface receptors, enhancing T cell adhesion, and promoting the cellular uptake of exosomes. Molecular docking and protein phosphorylation studies confirmed that actinators can modulate actin-binding proteins like cofilin. Actinators represent a class of actin-derived bioactive peptides with diverse applications in gene and immune therapies, drug delivery, and disease treatment.

## Teaser

Actinators are a class of bioactive peptides with applications in gene and immune therapies, drug delivery, and disease treatment.

## INTRODUCTION

Actin is a 43-kDa cytoskeletal protein that polymerizes to form actin filaments (F-actin), rigid and dynamic fibers ~7 nm in diameter and up to several micrometers in length (*1*–*3*). Actin filaments and their regulatory proteins, such as cofilin and the actin-related protein 2/3 complex (Arp2/3) (*4*), localize mostly beneath the cortex of the plasma membrane to form a meshwork with the properties of semisolid gels (*5*). This cortical actin provides mechanical support and determines cell shape; also, actin polymerization and depolymerization, the process of actin treadmilling, provide the driving force for cell migration (*6*, *7*). In addition, actin is involved in multiple biological processes such as cell adhesion (*8*), receptor cycling (*9*), endocytosis/exocytosis (*10*), and viral infection (*11*, *12*). In particular, in viral infection, cortical actin has been suggested to be a barrier that can impede viral entry and intracellular migration (*11*). The requirement for breaking the cortical actin barrier during viral infection is exemplified in HIV-1 latent infection of blood resting CD4 T cells; in this process, HIV-1 uses the chemokine coreceptor CXCR4 or CCR5 to trigger heterotrimeric guanine nucleotide–binding protein signaling, resulting in the activation of actin regulators, such as cofilin, to promote the actin dynamics necessary for viral entry and nuclear migration (*11*, *13*–*15*). Inhibition of HIV-mediated actin activity diminishes viral infection of blood CD4 T cells, whereas increasing actin dynamics promotes viral infection (*11*, *15*–*17*). The requirement for actin dynamics and cofilin activity in infection is shared by multiple viruses such as herpes simplex virus (*18*), measles virus (*19*, *20*), alphaherpesvirus (*21*), Zika virus (*22*), and wheat dwarf virus (*23*). Nevertheless, despite some capacity to trigger actin activity during viral entry, viral vectors frequently exhibit low transduction of cells in vitro and often require the use of highly concentrated particles to force viral entry (*17*, *24*–*26*). Thus, we attempted to develop novel technologies, on the basis of the cortical actin barrier theory (*11*), to facilitate viral vector transduction through promoting cortical actin dynamics. For this purpose, we designed several libraries of synthetic peptides using the amino acid sequence of human β-actin in hope of finding unique bioactive, actin-derived peptides that could interfere with actin-binding proteins (ABPs) to modulate actin dynamics. We performed screening and validation using HIV-1 infection of human CD4 T cells as a model. Here, we describe the discovery of six β-actin–derived peptides, collectively named actinator, that are biologically active and capable of modulating a variety of actin-related cellular functionalities such as cell attachment, surface receptor recycling, and cellular uptake of viral particles and extracellular vesicles (EVs). These actinators represent a previously unknown group of actin-derived bioactive peptides that have potential applications in drug delivery, gene and cell therapies, and disease treatment.

## RESULTS

### Design and screening of human β-actin–derived peptides

**Fig. 1.**
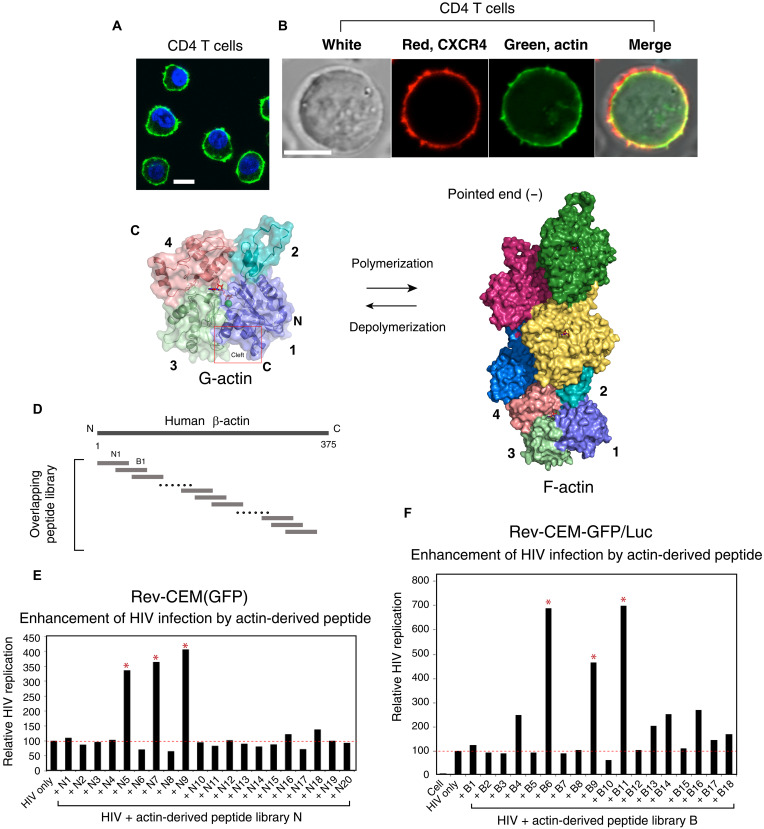
Design, synthesis, and screening of actin-derived bioactive peptides. (**A**) Human blood CD4 T cells were fixed and stained with FITC (fluorescein isothiocyanate)-phalloidin for F-actin and DAPI (4′,6-diamidino-2-phenylindole) for nuclear DNA. Scale bar, 5 µm. (**B**) Cells were also transfected with a LifeAct-GFP vector (green) and stained for surface CXCR4 (red). Scale bar, 5 µm. (**C**) Structure of G-actin and F-actin. The location of the four G-actin domains and the hydrophobic cleft are shown. (**D**) Schematic of actin-derived peptide libraries synthesized. (**E** and **F**) Screening for bioactive peptides for enhancing HIV-1 infection of indicator T cells. Rev-CEM(GFP) or Rev-CEM-GFP/Luc indicator T cells were pretreated with 10 μM of each peptide for 1 hour, infected with HIV-1 for 2 hours, washed twice with medium, and then cultured for 2 days in the absence of the peptide. HIV-1 replication was quantified by the flow cytometry of GFP^+^ cells (figs. S1 and S2). As a control, a sequence-scrambled peptide N20 was used.

**Fig. 2.**
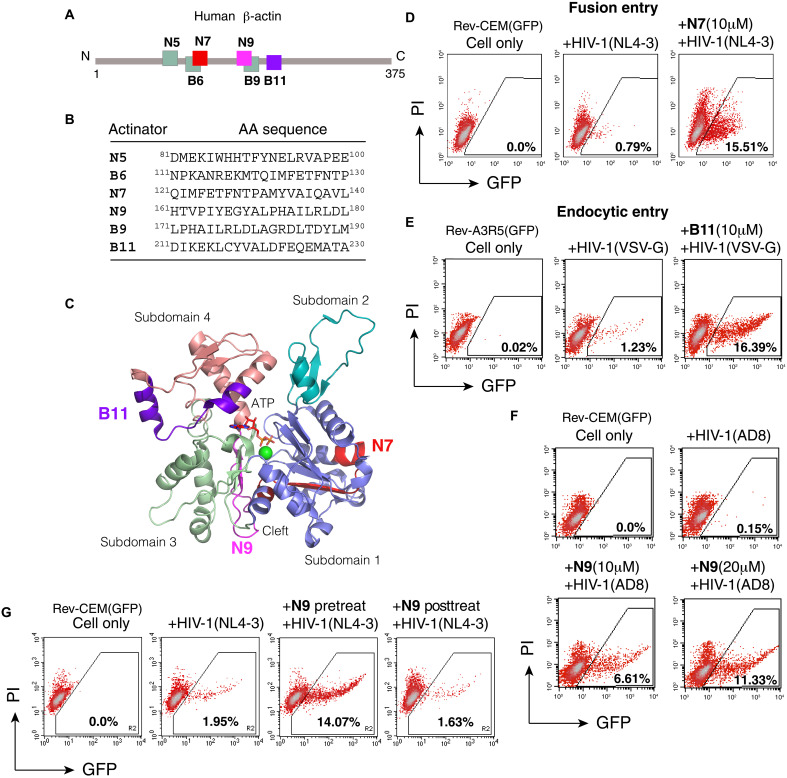
Discovery of actinators. (**A** and **B**) Locations (numeration from N to C terminus of human β-actin) and amino acid (AA) sequences of actinator peptides found through screening. (**C**) Locations of actinators N7, N9, and B11 on the structure of G-actin. ATP, adenosine 5′-triphosphate. (**D** and **E**) Enhancement of HIV-1 infection of Rev-dependent indicator T cells by N7 and VSV-G–pseudotyped HIV-1 infection by B11. Cells were pretreated with 10 μM of each peptide for 1 hour, infected with the viruses for 2 hours, washed twice with medium, and then cultured for 2 days in the absence of the peptides. GFP expression was quantified by flow cytometry. PI was used during flow cytometry to quantify viral replication only in live cells. (**F**) Actinator dosage–dependent enhancement of HIV-1(AD8) infection by N9. Cells were treated, infected, and analyzed similarly as described above. (**G**) The actinator N9 enhances HIV-1 infection only when used before HIV-1 infection but not after HIV-1 infection. Cells were treated with N9 for 1 hour either before adding HIV-1 (pretreat) or after HIV-1 infection for 2 hours (posttreat). Cells were washed and cultured for 2 days. GFP expression was quantified by flow cytometry.

In human CD4 T cells, cortical actin is localized beneath the plasma membrane and is one of the first intracellular structures that viruses engage with during receptor binding and viral entry (Fig. 1, A and B). Actin is extremely conserved (90% identity between yeast and humans) (*27*) and structurally organized into two major domains and four subdomains numbered 1 to 4 (Fig. 1C) (*28*). In monomeric actin (G-actin), two diametrically opposed clefts separate the two large domains; the larger cleft, between subdomains 2 and 4, constitutes the nucleotide-binding site, whereas the smaller hydrophobic cleft (*29*), between subdomains 1 and 3, is the interacting area for most ABPs including cofilin (*30*). ABPs are diverse, both structurally and functionally. Nevertheless, most ABPs share the common binding pocket around the hydrophobic cleft; other ABPs bind to filamentous actin (F-actin) along the side or at the barbed/pointed ends (*30*, *31*). On the basis of the actin structure and its interaction with ABPs, we speculated that certain actin-derived peptides may competitively affect the binding of ABPs to actin, thereby disturbing actin treadmilling and actin dynamics. Given the requirement of actin dynamics for viral infection, we also speculated that such actin peptides may affect viral infection through modulating actin activities. To test this hypothesis, we constructed several overlapping libraries of actin peptides of 10 to 20 amino acids (identical amino acid sequences to human β-actin fragments) that are linked to cell-penetrating peptides such as polyarginine or the HIV Tat-peptide for intracellular delivery (Fig. 1D) (*32*, *33*). We used HIV-1 infection as a system to detect possible effects on viral infection. The HIV-1 Rev-dependent green fluorescent protein (GFP) indicator CD4 T cell, Rev-CEM(GFP) or Rev-CEM-GFP/Luc (*34*), was used as the target cell to quantify HIV infection. Cells were briefly treated with each individual peptide for 1 hour and then infected with HIV-1 in the presence of the peptide for 2 hours. Both the virus and peptide were washed away, and HIV replication was quantified by GFP reporter expression at 2 to 3 days postinfection (Fig. 1, E and F, and figs. S1 and S2). Screening of the actin peptide libraries identified six top candidates (N5, N7, N9, B6, B9, and B11) that enhanced HIV infection without detectable cytotoxicity (Fig. 2, A and B). We named this group of bioactive peptides actinator on the basis of their capacity to enhance cellular susceptibility to HIV infection.

###  Actinators enhance HIV entry and infection

We chose actinators N7, N9, and B11 as examples for validation and detailed mechanistic studies (Fig. 2C). Actinator-mediated enhancement of viral infection requires its intracellular delivery, as removal of the cell-penetrating peptide from B11 diminished its capacity to enhance viral infection (fig. S3); the cell-penetrating peptide by itself without connecting to an actinator peptide was also not active for enhancing viral infection (e.g., N20) (Fig. 1E and fig. S1). In addition, an actinator does not promote nonspecific viral infection in the absence of virus-receptor interaction; when HIV-1 gp120 binding to CD4/CXCR4 was blocked by a neutralization antibody, VRC03, HIV-1 infection was completely diminished even in the presence of B11 (fig. S4). Nevertheless, actinator-mediated enhancement of HIV infection did not depend on HIV strains or subtypes, as both the CXCR4-using and CCR5-using HIV-1 strains, as well as HIV-2, were all enhanced (Fig. 2, D to F, and fig. S5). The range of enhancement varied, from 3- to 5-fold to as high as 20- to 30-fold (Fig. 2, D and E, and fig. S5), depending on particular viral strains, viral dosages, and types of target cells used. We also attempted to use an actinator to rescue primary viral isolates that are difficult to propagate in vitro; a primary HIV-2 isolate, CDC310072, was used for growth in human A3R5 CD4 T cells. Treatment of cells with the actinator B11 during infection successfully rescued HIV-2 CDC310072 replication, while in the absence of B11 treatment, viral replication was undetectable even 1 week after infection (fig. S5).

Actinator-mediated enhancement is also dosage-dependent, as we observed greater enhancement at 20 to 30 μM than at 10 μM (Fig. 2F). The enhancement can occur very rapidly, with even a 5-min pretreatment (fig. S6). However, only preinfection treatment, but not postinfection treatment, of cells with an actinator enhanced viral infection (Fig. 2G), suggesting that an actinator specifically enhances viral early steps such as viral entry and nuclear migration, which are known to require actin dynamics (*11*). Given that actinator-mediated enhancement can reach over 20-fold even in permissive T cell lines such as CEM-SS (Fig. 2D), these results suggest that, in some cases, more than 95% of infecting virions can be restricted by the cortical actin barrier and, furthermore, that this restriction can be alleviated by increasing actin dynamics through actin modulators such as an actinator.

**Fig. 3.**
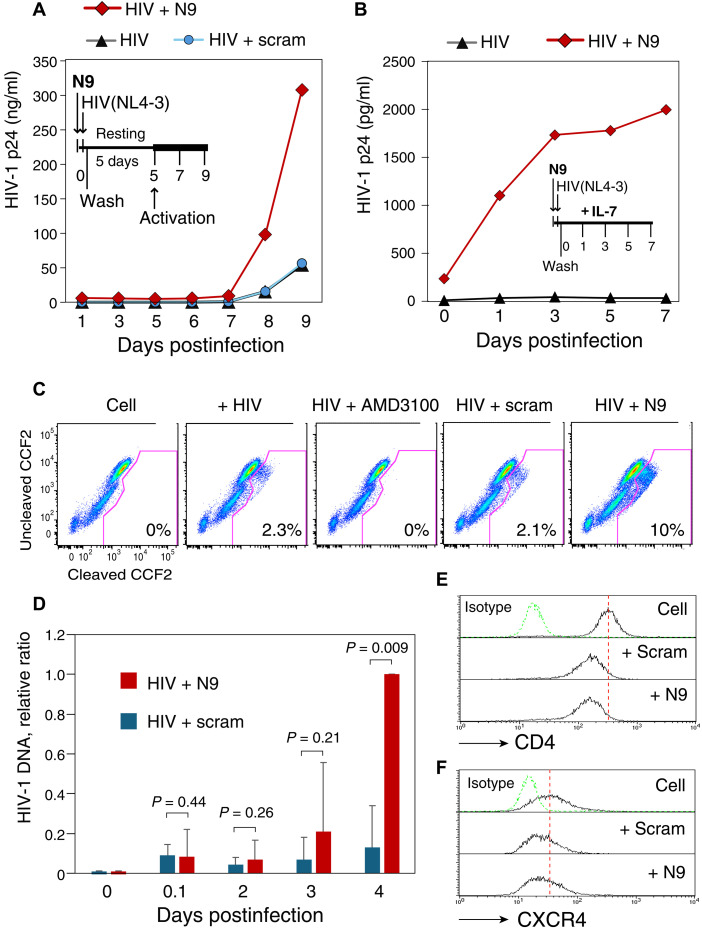
Actinator N9 enhances HIV-1 entry and latent infection of blood resting CD4 T cells. (**A** and **B**) N9 enhances HIV-1 latent infection of resting CD4 T cells. Cells were pretreated with N9 (10 μM) for 1 hour, infected with HIV-1 for 2 hours, washed twice with medium, cultured for 5 days, and then activated with anti–CD3/CD28 beads (A). In independent experiment repeats, infected cells were also cultured with IL-7 (1 ng/ml) without anti–CD3/CD28 activation (B). Shown is viral replication measured by p24 release. Experimental replicates are shown in fig. S5. (**C**) N9 enhances HIV-1 entry into resting T cells. Cells were pretreated with N9 (10 μM) for 1 hour and then infected with a reporter virus, HIV-1(BlaM-Vpr). As controls, cells were also pretreated with AMD3100 (10 μM) or a scrambled peptide (10 μM) and then infected. Viral entry was quantified by the flow cytometry analysis of cleaved CCF2. (**D**) N9 enhances viral entry as quantified by levels of intracellular HIV-1 DNA. The relative DNA copy numbers are plotted using averages [means ± standard deviation (SD)] from three independent infection experiments. *P* values were calculated using a paired *t* test. (**E** and **F**) Effects of N9 on the CD4 and CXCR4 receptors on CD4 T cells. Cells were treated with N9 (10 μM) for 1 hour, and the CD4 or CXCR4 receptor was stained with phycoerythrin-labeled anti-CD4 or anti-CXCR4 antibodies and analyzed by flow cytometry.

To further characterize the specific viral steps affected by an actinator, we followed HIV-1 latent infection of blood resting CD4 T cells, in which cortical actin has been shown to restrict viral latent infection (*11*). In this latent infection model, resting CD4 T cells were pretreated with or without N9 and then infected with HIV-1 for 5 days (Fig. 3A). Cells were subsequently activated with CD3/CD28 stimulation, and viral replication was quantified. We observed an enhancement of HIV-1 latent infection by N9. We also repeated the experiment by directly infecting CD4 T cells cultured in interleukin-7 (IL-7) without T cell activation (Fig. 3B) (*35*). Again, we observed a marked enhancement of HIV-1 latent infection by N9. The enhancement was observed in latent infection of resting T cells from multiple donors (fig. S5).

We further quantified the effects of N9 on HIV entry using the Vpr-based lactamase entry assay (*36*) and found an enhancement of viral entry in N9-treated CD4 T cells (Fig. 3C). The enhancement of viral entry was also confirmed by quantifying intracellular HIV-1 DNA synthesis (Fig. 3D). This enhancement was not attributed to T cell activation, as N9 did not induce the expression of T cell activation markers, including the CD25 and CD69 receptors (fig. S7); only phytohemagglutinin + IL-2 stimulation, but not actinator stimulation, led to the up-regulation of CD25 and CD69. Cell cycle analysis by intracellular DNA/RNA staining further confirmed that N9 does not trigger T cell activation (fig. S7). We also quantified the effects of N9 on the surface expression of HIV entry receptors and found that N9 did not up-regulate CD4 or CXCR4 (Fig. 3, E and F). These results suggest that N9 likely triggered transient cellular actin activities during viral entry to enhance the cellular uptake of viral particles. Our previous studies have shown that enhancing actin dynamics can promote viral entry and nuclear migration (*11*).

### Actinator B11 promotes retro- and lentiviral vector transduction and cellular uptake of EVs

**Fig. 4.**
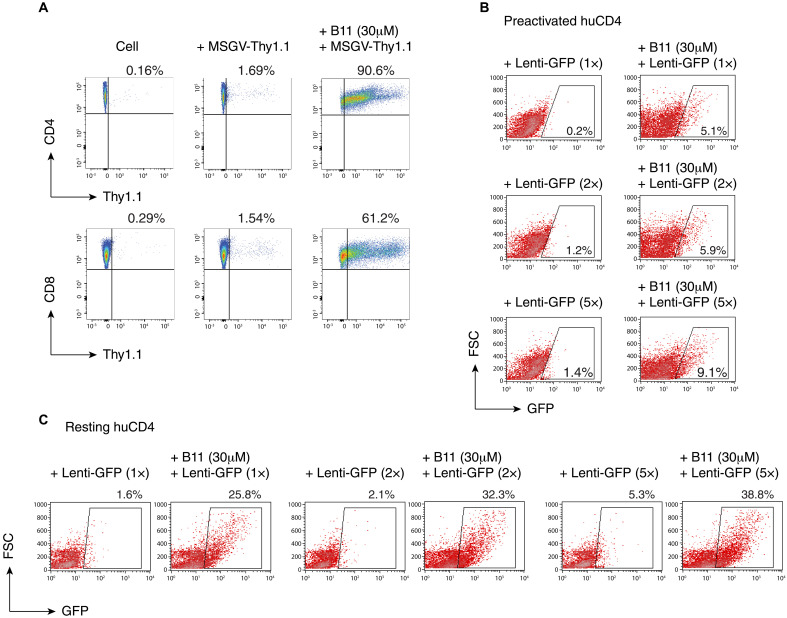
Actinator B11 enhances viral vector transduction of primary mouse and human T cells. (**A**) The actinator B11 enhances retroviral vector transduction of primary mouse CD4 and CD8 T cells. Mouse CD4 and CD8 T cells were purified from lymph nodes and spleen and then activated with anti–CD3/CD28 antibodies. Cells were pretreated or not with B11 for 1 hour and then infected with MSGV-Thy1.1 retroviral particles for 4 hours. Cells were washed with medium, cultured for 24 hours, and analyzed for Thy-1.1 expression with flow cytometry. (**B**) The actinator B11 enhances lentiviral vector transduction of preactivated human blood CD4 T cells. Human CD4 T cells were purified from peripheral blood and then activated and expanded with anti–CD3/CD28 antibodies for 3 days (four beads per cell). Cells were pretreated with B11 for 30 min and then infected with various doses (1× to 5×) of unconcentrated Lenti-GFP lentiviral particles for 2 hours. Cells were washed with medium, cultured for 48 hours, and analyzed for GFP expression with flow cytometry. FSC, forward scatter. (**C**) The actinator B11 enhances lentiviral vector transduction of human resting CD4 T cells. Human CD4 T cells were purified from peripheral blood and then cultured overnight. Cells were pretreated with B11 for 30 min and then infected with various doses of unconcentrated Lenti-GFP lentiviral particles for 2 hours. Cells were washed with medium and activated immediately with anti–CD3/CD28 antibodies for 2 days. Cells were analyzed for GFP expression with flow cytometry.

Chimeric antigen receptor (CAR) T cell therapies involve in vitro transduction and engineering of patients’ immune T cells with tools such as retroviral vectors. We further tested the capacity of actinators to enhance the retroviral transduction of mouse primary CD4 and CD8 T cells. Mouse T cells were first purified and then activated with anti–CD3/CD28 antibodies. Cells were then transduced with a retroviral reporter virus, vMSGV-Thy1.1. As shown in Fig. 4A, in the absence of the actinator B11, unconcentrated vMSGV-Thy1.1 particles transduced 1.69 and 1.54% of CD4 and CD8 T cells, respectively. Treatment of CD4 and CD8 T cells with B11 markedly enhanced the viral transduction rate to 90.69 and 61.2%, respectively (Fig. 4A). Similar enhancements were also observed in independent experimental repeats (fig. S8), and an ~20- to 30-fold enhancement was observed. We further tested the ability of B11 to enhance the transduction of human blood CD4 T cells with lentiviral particles pseudotyped with the VSV-G (vesicular stomatitis virus glycoprotein) envelope. B11 also enhanced lentiviral particle transduction of resting CD4 T cells and CD3/CD28–preactivated CD4 T cells (Fig. 4, B and C), demonstrating its potential application to enhance lentiviral vector delivery of therapeutic genes into CAR T cells.

**Fig. 5.**
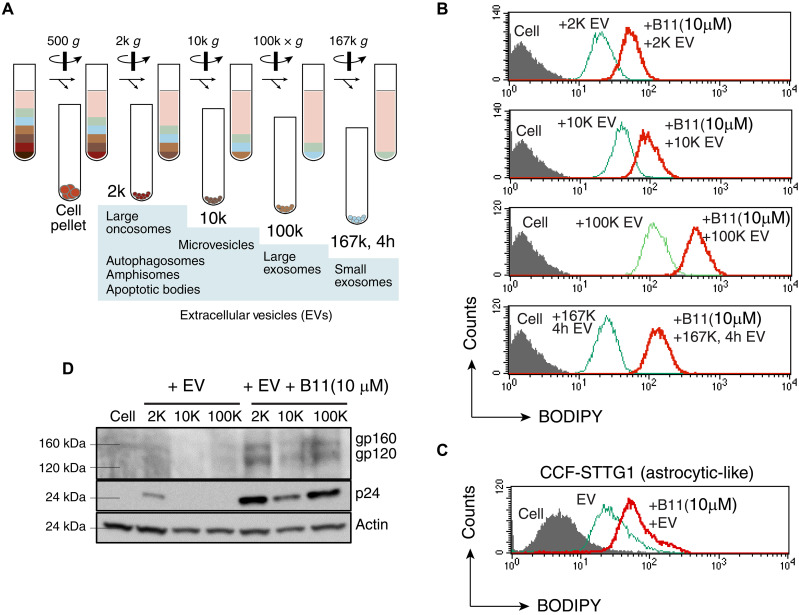
Actinator B11 promotes the cellular uptake of EVs. (**A**) Schematic overview of EV separation procedures using differential centrifugation and ultracentrifugation. h, hour. (**B** and **C**) B11 enhances the cellular uptake of EVs released from HIV^+^ J1.1 T cells. EVs purified in (A) were labeled with BODIPY and then incubated with recipient cells, either Jurkat (B) or CCF-STTG1 (C) for uptake assays. Cells were pretreated or not with B11 for 1 hour and then incubated with BODIPY-labeled EVs for 1 hour, cultured for 24 hours, washed, and then analyzed by flow cytometry. (**D**) Western blot quantification of B11-mediated enhancement of cellular uptake of EVs released from HIV^+^ J1.1 T cells. Recipient Jurkat T cells were pretreated or not with B11 for 1 hour and then incubated with EV for 1 hour, cultured for 24 hours, washed, lysed, and then analyzed with Western blotting using primary antibodies against HIV-1 Env, HIV-1 p24, or cellular actin. Horseradish peroxidase–conjugated goat anti-mouse secondary antibodies were used to detect proteins using chemiluminescence.

Intracellular communication frequently involves cellular release and uptake of EVs, which are a heterogeneous population of membrane vesicles that includes exosomes, microvesicles, and apoptotic bodies (*37*, *38*). EVs are important vehicles for cell-cell communication and for the intercellular delivery of therapeutic genes and drugs (*39*). We investigated whether an actinator enhances the cellular uptake of EVs. As shown in Fig. 5A, various EVs were produced from HIV^+^ Jurkat T cells (J1.1) (*40*) and purified through differential ultraspeed gradient centrifugation (from 2000*g* to 167,000*g*). The 2000*g* fraction contains large vesicles such as autophagosomes and apoptotic bodies, the 10,000*g* fraction contains mostly microvesicles, the 100,000*g* fraction contains large exosomes, and the 167,000*g*/4-hour fraction contains small exosomes. These purified EVs were fluorescently labeled and then delivered to human Jurkat T cells or astrocyte-like CCF-STTG1 cells in the presence or absence of the actinator B11. We observed the enhancement of cellular uptake of these HIV^+^ EVs when cells were treated with B11 (Fig. 5, B and C). In particular, the greatest enhancement was observed in the cellular uptake of small exosomes, with an ~10-fold increase in the peak fluorescence intensity. We repeated the experiment and used Western blot to confirm the enhanced intracellular delivery of HIV-1 proteins via EVs and observed the substantial enhancement of EV uptake by Jurkat cells in the presence of B11 (Fig. 5D). These results suggest that cortical actin may also be involved in the entry of certain EVs that may use cellular receptors for entry and that actinator B11 is capable of enhancing the cellular uptake of these EVs, likely through promoting actin activity as seen in the enhancement of HIV infection.

### Actinator molecular docking and cofilin phosphorylation studies

**Fig. 6.**
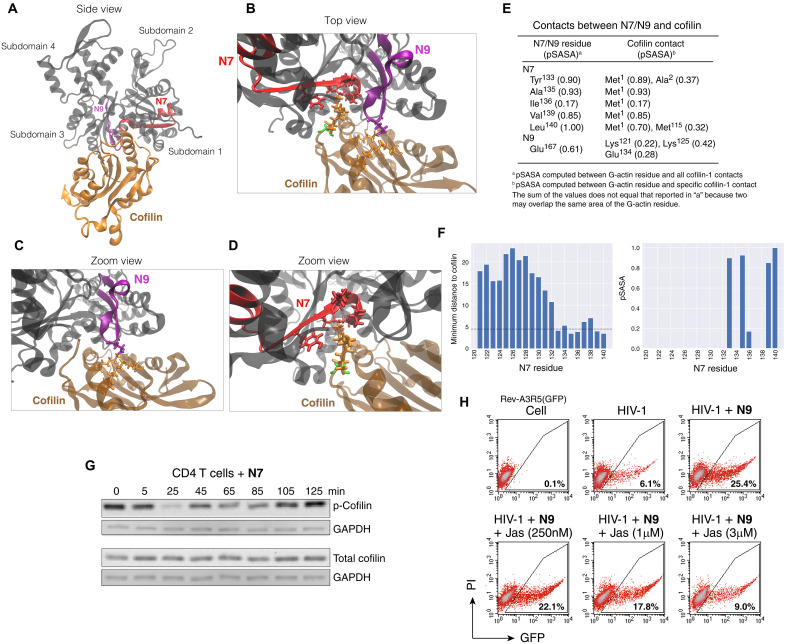
Interaction of actinators N7 and N9 with cofilin. (**A** to **D**) Cofilin (gold) bound to G-actin (gray) predicted from AlphaFold. N7 and N9 peptides are colored in red and purple, respectively (A). The amino acid contacts between N7 or N9 with cofilin are shown in licorice representation. Ser^3^ on cofilin is shown in green (B and D). (**E** and **F**) pSASA computed contacts between N7/N9 and cofilin. Both overall and specific residue contacts are calculated (F), and the distance of N7 residues to cofilin is also computed (F). (**G**) N7-mediated cofilin activation in resting CD4 T cells was quantified with Western blotting using an anti–p-cofilin antibody. Cells were treated with N7 (10 μM) for various times and analyzed using antibodies against p-cofilin. The blot was also probed with antibodies against total cofilin or GAPDH. (**H**) Jas inhibits N9-mediated enhancement of HIV-1 infection. Cells were pre-treated with N9 (10 μM) or N9 plus different doses of Jas for 1 hour and then infected with HIV-1 for 2 hours. Cells were washed and cultured for 2 days. GFP expression was quantified by flow cytometry.

Structurally, the actinator peptides are located mostly in regions surrounding the edge of the hydrophobic cleft between subdomains 1 and 3, except for the actinator B11, which is localized to the α-helical loop on the edge of subdomain 4 (Fig. 2C). The regions of N7 and N9 lie on the edge of the hydrophobic pocket that overlaps the binding regions of ABPs such as cofilin and thus may alter their interaction with actin; actinator-mediated enhancement of viral entry likely results from interfering with ABPs, leading to a transient increase in actin treadmilling. Actin dynamics are mainly regulated by ABPs such as cofilin, which depolymerizes actin filaments (*41*). We performed detailed molecular docking studies to determine the potential impacts of actinators N7 and N9 on cofilin interaction with actin. As shown in Fig. 6A, cofilin is docked to the G-actin hydrophobic cleft located between subdomains 1 and 3, engaging 10 of the 11 actin residues lining this cleft (*30*). We measured specific contacts between G-actin and cofilin residues when the distance between any two of their nonhydrogen atoms was less than 4.5 Å. There are, in total, 73 contacts between G-actin and cofilin, and more than 60% of these contacts involve at least one hydrophobic amino acid. In particular, the cofilin hydrophobic N-terminal residues Met^1^ and Ala^2^ are inserted into the actin hydrophobic cleft (Fig. 6, A to D). These two hydrophobic residues are in contact with actin residues Tyr^133^, Ala^135^, Ile^136^, Val^139^, Leu^140^, Tyr^143^, Leu^346^, Phe^352^, Met^355^, and Phe^375^. Half of these contacts arise from the residues contained within N7 (Fig. 6E). Therefore, N7 residues represent a critical hydrophobic core that is partially responsible for stabilizing the cofilin N terminus in the hydrophobic cleft. The N9 residue Glu^167^ is also in contact with cofilin residues Lys^121^, Lys^125^, and Glu^134^ (Fig. 6E), and it is possible that the formation of these salt bridges may help to stabilize the complex. In summary, both N7 and N9 residues are involved in interactions with cofilin, although the nature of these interactions is different: N7 residues create a hydrophobic core in the G-actin hydrophobic cleft that supports cofilin docking, whereas N9 residues promote electrostatic interactions that provide an additional “lock” on the bound state.

We further measured the relative strength of contacts between N7 or N9 residues and cofilin and computed the solvent-accessible surface area (SASA). We computed SASA in VMD (*42*) using a probe radius of 1.4 Å. Specifically, we calculated the percentage of an N7 or N9 residue’s SASA in the unbound state (with cofilin removed) when the residue is occupied by cofilin (pSASA). As shown in Fig. 6E, all N7 residues that form contacts, excluding Ile^136^, form substantial interactions when pSASA >0.8. In addition, in all of these cases, cofilin Met^1^ is the primary contact residue (Fig. 6, E and F). Contacts between N9 and cofilin residues are marginally less significant (pSASA ≈ 0.6), and no specific contact occupies more than 42% of the N9 Glu^167^ SASA (Fig. 6, E and F). These data reinforce that cofilin interactions with N7 residues represent a critical step in the formation of their interface, whereas N9 overall plays a supporting role to the docking process.

**Fig. 7.**
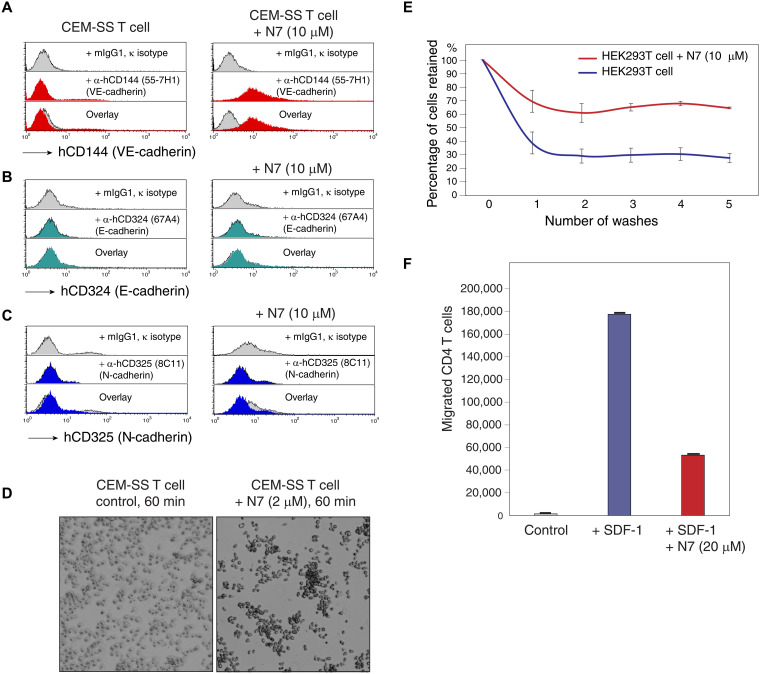
The actinator N7 modulates cell surface VE-cadherin, promotes cell-cell and cell-matrix attachments, and inhibits transformed T cell chemotaxis. (**A** to **C**) N7 modulates surface staining of CD144 (VE-cadherin) but not CD324 (E-cadherin) or CD325 (N-cadherin). CEM-SS T cells were treated with N7 for 30 min, stained with labeled anti-CD144 (A), anti-CD324 (B), or anti-CD325 (C) antibodies, and then analyzed with flow cytometry. The experiments were independently repeated more than three times. (**D**) N7 promotes cell-cell attachment. CEM-SS CD4 T cells were treated or not with N7 (2 μM) for 60 min at 37°C. Cells were imaged using light microscopy. (**E**) N7 promotes cell-matrix attachment. HEK293T cells, grown on a cell culture–treated 96-well plate overnight, were pretreated with N7 (N7, 10 μM) or not (control) for 1 hour at 37°C. Cells were then repeatedly washed with PBS and then stained with crystal violet for 20 min. Cells retained were quantified in solubilization buffer for crystal violet absorbance (560 nm) using GloMax. Data represent the average (means ± SD) of three assay triplicates. (**F**) N7 inhibits transformed T cell chemotaxis. A3R5.7 CD4 T cells were pretreated with N7 (20 μM) for 30 min at 37°C. Cells were then placed in the upper chambers of a transwell plate assayed for migration toward SDF-1 (40 ng/ml). Results are expressed as the number of migrating cells. Error bars represent SDs from three assay quantifications (means ± SD).

Cofilin Ser^3^ phosphorylation is a key regulatory mechanism of cofilin activity (*43*). When cofilin is complexed with G-actin, the Ser^3^ side chain is not solvent exposed [SASA is 49.2 Å^2^, whereas in the reference triplet state, SASA is 2.6-fold higher (*44*)] and is instead directed toward the cofilin interior (Fig. 6D). Ser^3^ phosphorylation, which is known to inhibit interactions between cofilin and actin (*45*), is therefore expected to result in a rearrangement of the cofilin backbone and to alter its interaction with actin. On the basis of the docking studies and given the close contacts of N7 residues with cofilin Met^1^ and Ala^2^, we speculated that N7 likely affects cofilin Ser^3^ phosphorylation (Fig. 6D). To experimentally validate the docking studies, we examined the effects of N7 on the activation of cofilin in CD4 T cells. As shown in Fig. 6G, treatment of blood resting CD4 T cells with N7 led to a brief, marked activation of cofilin, occurring 5 to 25 min after the addition of N7. The cycles of cofilin dephosphorylation and phosphorylation, a marker of actin treadmilling, lasted for about 2 hours. These results suggest that N7 enhances viral infection likely through transient promotion of actin dynamics by modulating ABPs. In addition to triggering cofilin activation, N7 was found to modulate cell surface receptors such as VE-cadherin (Fig. 7A and fig. S9) and promote cell-cell adhesion (Fig. 7, D and E). N7 was also found to inhibit T cell chemotaxis at 20 μM (Fig. 7F), a process driven by actin treadmilling regulated by Arp2/3 and cofilin (*46*). These properties of N7 are likely linked to its ability to modulate the cortical actin dynamics via cofilin and other ABPs.

To further confirm that the actinator-mediated enhancement of viral infection is related to actin dynamics, we treated CD4 T cells with an actin inhibitor, jasplakinolide (Jas), which binds to F-actin and prevents actin treadmilling (*47*). As shown in Fig. 6H, treatment of cells with Jas largely abolished the actinator-mediated enhancement of viral infection, confirming the role of actin dynamics in the actinator-mediated enhancement of viral infection.

## DISCUSSION

Here, we describe the design, screening, and discovery of actinators. Given that a majority of the actin-derived peptides that we screened did not demonstrate bioactivity in our assays, actinator peptides represent unique actin sequences or domains that may interact or interfere with specific sets of ABPs, thereby affecting the actin dynamics needed for viral entry. Structurally, monomeric actin is rigid for spontaneous actin polymerization, whereas actin filaments, once formed, have limited internal regions accessible for ABPs. Four of the six actinator peptides (B6, N7, N9, and B9) are actin amino acid sequences surrounding the hydrophobic cleft between subdomains 1 and 3, where most ABPs, such as cofilin, profilin, and gelsolin, bind to actin filaments (*4*). The other two actinator peptides, B11 and N5, are α-helical loops on the external edge of subdomains 4 and 2, respectively, which likely overlap other ABP binding sites or are involved in actin-actin interactions. It is possible that these actinator sequences are involved in ABP interactions either fully or partially (Fig. 6), and actinator-mediated bioactivity may result from interference with actin-ABP interactions, leading to the transitory modulation of actin dynamics.

The binding of peptides derived from actin to cofilin has previously been demonstrated in biochemical binding assays. Using an array of actin-derived peptides cross-linked to cellulose membranes, Mannherz *et al.* (*48*) have identified five sequence stretches of actin capable of binding cofilin (actin peptides with sequence positions 34 to 44, 52 to 62, 146 to 153, 328 to 338, and 346 to 353). Kamal *et al.* (*49*) also explored the ability of actin-derived peptides to bind to cofilin and protect it from oxidative modification. The authors identified nine peptides that confer strong protection against modification (actin peptides with sequence positions 1 to 18, 51 to 61, 63 to 68, 85 to 95, 96 to 113, 118 to 125, 197 to 206, 239 to 254, and 360 to 372) and eight peptides with moderate protection (actin peptides with sequence positions 40 to 50, 69 to 84, 119 to 147, 148 to 177, 184 to 191, 260 to 270, 292 to 312, and 316 to 326). In addition, Hori and Morita (*50*) have shown that the actin-derived peptide from sequence position 179 to 198 affects actin polymerization assays by putatively interfering with actin-actin interactions. On the basis of these previous studies and ours, several possible mechanisms can be speculated regarding how actinators affect actin dynamics. First, actinators such as B6, N7, and N9 may interfere with cofilin binding to actin; the actin peptides from positions 118 to 125, 119 to 147, and 148 to 177, identified by Kamal *et al.* (*49*) for cofilin binding, partially overlap the sequences of our actinators B6 (positions 111 to 130), N7 (positions 121 to 140), and N9 (positions 161 to 180). Second, actinators such as B9 may interfere with actin polymerization by interfering with actin-actin interactions; the actin peptide 179 to 198, identified by Hori and Morita (*50*), also partially overlaps the sequence of the actinator B9 (positions 171 to 190).

It has been known that phosphorylated cofilin (p-cofilin) binds poorly to both G-actin (monomers) and F-actin (filaments), but dephosphorylated cofilin is highly active and preferentially binds to F-actin, causing filament severing and disassembly. p-Cofilin acts as an inactive storage form; it must be dephosphorylated to become active and interact with F-actin to promote actin turnover (*51*). It is possible that actinators such as N7 interfere with active cofilin binding to F-actin, changing the balance between p-cofilin and active cofilin in cells. This may alter the rate of actin depolymerization at the pointed end, resulting in a transient increase in actin polymerization at the barbed end, which can be mediated by the default activities of other actin regulators such as Arp2/3 (*52*). We have observed that the treatment of T cells with N7 triggers cycles of cofilin dephosphorylation and phosphorylation, beginning at 5 min and lasting for ~2 hours (Fig. 6G).

Cofilin-mediated actin dynamics have been shown to promote viral entry and intracellular migration (*11*, *13*, *17*). In addition, it has been known that cofilin dephosphorylation promotes actin polymerization; the formation of F-actin–dependent positive feedback loops, including Ras-PI3K (phosphatidylinositol 3-kinase) signaling, is a hallmark of cellular activity and sensitivity (*53*–*55*). Consequently, this pathway contributes to micropinocytosis and phagocytosis, the two principal mechanisms by which cells internalize fluid-phase nutrients and solid particles such as viruses and exosomes (*56*–*59*).

Although actinators enhance viral entry, for individual actinator peptides, the level of enhancement varies, depending on both cell types and the modes of viral entry. For example, actinators achieved greater enhancements of infection in primary resting CD4 T cells than in transformed T cells (Fig. 4C and fig. S10) and for viruses entering cells directly through plasma membrane fusion. In addition, while all actinator peptides greatly enhance HIV entry, which is through membrane fusion (*60*), only B11, but not N9 and N7, enhanced VSV-G–pseudotyped lentivirus infection (Fig. 2E and fig. S11), which uses endocytic entry (*61*). B11 was also found to enhance the cellular uptake of nonenveloped viral particles, such as adeno-associated virus (AAV) (fig. S12), and exosomes that enter cells via various mechanisms (Fig. 5) (*62*). It is possible that the cortical actin restriction may be more pronounced in resting T cells in the absence of cell cycle–mediated cortical actin activity (*11*). In addition, different sets of ABPs may be involved in different modes of viral entry, either through fusion or endocytosis, as reflected in the selective bioactivities of individual actinator peptides. For instance, it has been shown that HIV-1 fusion and entry into resting T cells require cofilin activation (*11*), whereas clathrin-mediated endocytosis appears to be linked to Arp2/3–mediated actin polymerization (*63*). The LIM kinase inhibitor R10015, which blocks cofilin phosphorylation, inhibits HIV infection but not VSV-G–pseudotyped HIV-1 entry (*16*), demonstrating the different impacts of cofilin on viral entry pathways via fusion versus endocytosis.

The functional diversity of ABPs likely leads to the differences in bioactivities of individual actinator peptides. For example, while N9 did not modulate surface receptors (e.g., CD4 and CXCR4; Fig. 3, E and F) in our screening, N7 nonspecifically enhanced surface staining of multiple surface receptors, including CD144 (VE-cadherin) but not CD324 (E-cadherin) or CD325 (N-cadherin) (Fig. 7, A to C, and fig. S9). N7 also promotes cell-cell (Fig. 7D) and cell-matrix adhesion (Fig. 7E), a unique property not shared with N9 or B11. Certainly, both cell-cell adhesion and cell-matrix adhesion involve actin cytoskeleton and specific sets of ABPs (*64*). The ability of N7 to promote cell adhesion may be related to its capacity to modulate VE-cadherin and/or other surface adhesin molecules (Fig. 7A) (*65*). In addition, N7 was found to inhibit transformed CD4 T cell chemotaxis at 20 μM (Fig. 7F), while B11 minimally affects T cell migration at the same dosage. We also attempted to study possible synergistic effects among actinator peptides. However, when peptides B11 and N7 were used in combination, we did not observe the additive enhancement of viral infection (fig. S13). It is possible that actin dynamics are highly regulated, and simultaneous modulation of multiple actin regulators may not always lead to a synergistical biological process.

Actin plays a crucial role in a variety of biological processes (*30*). Actin-derived bioactive peptides, such as the actinator peptides found in this study, represent a previously unknown class of actin-modulating peptides that can affect fundamental actin biology and actin-related biological processes. Thus, actinators are expected to represent a useful tool in various applications, such as gene therapy, cell therapy, intracellular drug delivery, and the treatment of actin-related diseases. For example, CAR T cell therapies have been effectively used in clinical settings to treat multiple malignancies (*66*) and autoimmune diseases (*67*). Nevertheless, the ex vivo production of CAR-expressing T cells typically depends on viral transduction or CRISPR-based genome editing, which presents several challenges, such as low transduction efficiency, inadequate T cell expansion, and lengthy manufacturing times (*68*). High concentrations of lentiviral particles are often necessary to ensure viral entry through VSV-G–mediated endocytosis (*69*). However, exposing T cells to high amounts of VSV-G–pseudotyped particles can lead to cytotoxic effects and hinder T cell functionality (*70*). The use of actinators can alleviate the requirement for high levels of particle concentration and thus lessen cytotoxicity. Our studies indicate that nonconcentrated retro- and lentiviral particles can achieve high transduction efficiency with the assistance of actinators (Fig. 4). Moreover, we have shown that actinators can facilitate lentiviral particle transduction of resting CD4 T cells (Fig. 4C), even though the VSV-G–mediated endocytic pathway is not effective in resting T cells (*61*, *71*). Immediate activation or cytokine treatment (*35*) of B11-primed resting T cells after lentiviral transduction can facilitate the successful transduction of a large number of T cells without prior T cell activation (Fig. 4C). Potentially, direct infection of resting T cells can have numerous benefits. First, extensive activation of T cells for transduction and expansion can lead to progressive differentiation toward effector cells and loss of sustained antileukemic activity (*72*, *73*). Transducing resting T cells and avoiding extensive ex vivo activation might result in less differentiated CAR T cells, which could potentially offer enhanced persistence and functionality in vivo (*74*). Studies have shown that CAR T cells produced from less differentiated (naive and memory) T cell subsets demonstrate better expansion and prolonged antitumor responses (*73*). Second, high levels of T cell activation and proliferation can induce activation-induced cell death (*75*), which can limit T cell persistence. Transduced resting T cells, upon transduction and infusion, might be less prone to early exhaustion and activation-induced cell death, potentially leading to more robust CAR T cell expansion and persistence within the patient. Furthermore, direct transduction of nonactivated T cells might also reduce the required ex vivo T cell expansion time, potentially simplifying the process of CAR T manufacturing protocols (*76*). While the actinator B11 exhibits slight transient inhibition of the T cell activation marker CD25, this effect diminishes after 6 hours of B11 treatment (fig. S14). In addition, we quantified effects of B11 on other markers of T cell activity and functionality, including the expression of CD69, CD107a, IFN-γ (interferon-γ), and TNF-α (tumor necrosis factor–α) after T cell activation. We did not observe significant inhibition of these markers, either in CD4 or in CD8 T cells (fig. S15).

In addition to ex vivo CAR T cell generation, in vivo CAR T cell generation offers an alternative by eliminating the complexities of ex vivo manufacturing, allowing for direct engineering and expansion of CAR T cells in patients. Recent studies using lipid nanoparticles have yielded promising outcomes in animal models for both cancer and autoimmune diseases (*77*). Likewise, in vivo CAR T cell generation, strategies using lentiviral vectors have shown effectiveness in mice (*78*, *79*). Despite these advancements, viral vector transduction efficiency remains a challenge for cell-based immunotherapies (*78*). Given that actinator peptides consist of native actin sequences and are nonimmunogenic, they hold potential for facilitating in vivo CAR T development.

There are several limitations to our studies. First, the enhancement of viral transduction by actinator peptides primarily relies on their ability to stimulate cortical actin activities. It is important to note that the enhancement of viral transduction can also be achieved by promoting viral particle attachment to target cells, suggesting that an actinator may be combined with these technologies. In addition, the use of actinators requires pretreatment of target cells before transduction, introducing an extra step in the process. Future studies should focus on incorporating the actinator directly into the viral particle production process to eliminate this additional step. Moreover, this study does not address potential in vivo impacts of actinators on the functionalities of target cells, especially CAR T cells. Persistent stimulation of actin activity can influence T cell functions. However, low levels or short-term disturbances of the actin cytoskeleton should be tolerable (fig. S15). Despite this, further research is necessary to explore these critical questions related to the potential in vivo effects of actinators.

## MATERIALS AND METHODS

### Cells and cell lines

All protocols involving human subjects were reviewed and approved by the George Mason University Institutional Review Board (protocol nos. 7232 and 1484400). All participants provided written informed consent. Participants enrolled in this study were first evaluated for HIV serology and CD4 T cell counts. Criteria for exclusion of subjects include HIV-1 infection and low CD4 counts. Peripheral blood (100 to 150 ml) was drawn from participants and used to purify peripheral blood mononuclear cells and CD4 T cells, as described previously (*11*).

Human embryonic kidney (HEK) 293T cells (American Type Culture Collection) were cultured in Dulbecco’s modified Eagle’s medium (high glucose) (Thermo Fisher Scientific) supplemented with l-glutamine/minimum essential medium-nonessential amino acids/sodium pyruvate (Thermo Fisher Scientific) and fetal bovine serum (FBS) (10%). CEM-SS [National Institutes of Health (NIH) HIV Reagent Program], Rev-CEM(GFP) (Virongy Biosciences), Rev-CEM-GFP/Luc (Virongy Biosciences), and Rev-A3R5-GFP (Virongy Biosciences) were cultured in RPMI 1640 medium (Thermo Fisher Scientific) supplemented with 10% FBS. J1.1 (NIH HIV Reagent program) cell culture was grown in RPMI 1640 medium supplemented with EV-depleted FBS (100,000*g* centrifugation for 90 min) for 5 days. Human blood resting CD4 T cells were purified from peripheral blood by two rounds of negative selection, as previously described (*11*). Briefly, for the first round of depletion, monoclonal antibodies against human CD14, CD56, HLA-DR, DP, DQ (BD Biosciences) were used. For the second round of depletion, monoclonal antibodies against human CD8, CD11b, and CD19 (BD Biosciences) were used. Antibody-bound cells were depleted by using Dynabeads Pan Mouse IgG (immunoglobulin G; Thermo Fisher Scientific). Purified cells were cultured in RPMI 1640 medium supplemented with 10% heat-inactivated FBS (Thermo Fisher Scientific), penicillin (50 U/ml) (Thermo Fisher Scientific), and streptomycin (50 μg/ml) (Thermo Fisher Scientific). Cells were rested overnight before infection or treatment. In certain experiments, IL-7 (1 ng/ml) was added every 2 days. Cell activation was performed using anti-CD3 and anti-CD28 antibody–conjugated magnetic beads at four beads per cell, as described previously (*11*). Briefly, 10 μg of monoclonal antibodies against human CD3 (clone UCHT1) (BD Biosciences) and 10 μg of monoclonal antibodies against human CD28 (clone CD28.2) (BD Biosciences) were conjugated to 4 × 10^8^ Dynabeads Pan Mouse IgG (Thermo Fisher Scientific) for 30 min at room temperature. Magnetic beads were washed with phosphate-buffered saline (PBS) and resuspended in 1 ml of PBS and 0.1% bovine serum albumin (BSA).

Mouse lymph nodes and spleen from C57BL/6J mice were harvested, and cells were isolated using a Falcon 40-μm cell strainer (Thermo Fisher Scientific) to mash organs for cell extraction. The resulting mash was washed with cold PBS and centrifuged at 1200 rpm and 4°C for 5 min to pellet cells. Cells were resuspended in PBS containing 2% FBS and 1 mM EDTA at 1 × 10^8^ cells/ml. Mouse T cells were isolated using an EasySep Mouse T Cell Isolation Kit (Stemcell) as recommended by the manufacturer. Purified cells were cultured in RPMI 1640 medium supplemented with 10% heat-inactivated FBS (Thermo Fisher Scientific) (1 × 10^6^ cells/ml). For T cell activation, a soluble anti-CD28 antibody (clone 37.51, Miltenyi Biotec) (1 μg/ml) and recombinant mIL-2 (PeproTech) (50 U/ml) were added into the cell culture medium, and cells were cultured in a plate precoated with an anti-CD3e antibody (clone 145-2C11, Miltenyi Biotec) (4 μg/ml) for 2 days at 37°C.

### Virus preparation and infection of cells

Virus stocks of HIV-1(NL4-3), HIV-1(AD8), BlaM-Vpr containing HIV-1(NL4–3), and VSV-G–pseudotyped HIV were prepared as described previously (*11*, *61*). Briefly, HEK293T cells were transfected using Lipofectamine (Thermo Fisher Scientific) or the EZ-Fectin DNA Transfection kit (Virongy Biosciences) with cloned proviral DNA. The supernatant was harvested at 48 hours and filtered through a 0.45-μm nitrocellulose membrane. Levels of p24 in the viral supernatant were measured with enzyme-linked immunosorbent assay (ELISA) using an in-house ELISA kit. HIV-2 CDC310072 was obtained from NIH HIV Reagent Program. Unconcentrated Lenti-GFP [p24 (~100 ng/ml)] was provided by Virongy Biosciences.

For HIV infection of Rev-dependent indicator T cells, Rev-CEM(GFP), Rev-CEM-GFP/Luc, and Rev-A3R5-GFP (Virongy Biosciences) (*34*, *80*), cells (2 × 10^5^/0.1 ml) were pretreated or not with individual actinator peptides, which were synthesized (with polyarginine or the Tat-peptide added at the N terminus during peptide synthesis) by Bio-Synthesis (Lewisville, US), Scilight Peptide (Beijing, China), or GL Biochem (Shanghai, China). Following actinator pretreatment, HIV and an additional amount of the actinator were added to maintain the actinator concentration. Cells were infected for 2 hours at 37°C, washed twice with medium to remove the unbound virus and actinator, and then resuspended in fresh medium (2 × 10^5^/ml) and cultured for 48 to 72 hours. HIV infection was measured by flow cytometry (FACSCalibur, BD Biosciences) for GFP expression. To exclude drug cytotoxicity, propidium iodide (PI) (2 μg/ml, Fluka) was added into the cell suspension before flow cytometry, and only the viable cell population (PI negative) was quantified for GFP expression. For infection of blood resting CD4 T cells, cells (1 × 10^6^/ml) were pretreated or not with an actinator. HIV and an additional amount of the actinator were added to maintain the actinator concentration. Cells were infected for 2 hours at 37°C, washed twice with medium to remove the unbound virus and actinator, then resuspended in fresh medium (1 × 10^6^/ml) and cultured for 5 days without stimulation, and lastly activated with anti–CD3/CD28 magnetic beads at four beads per cell, as described above. In the instances where cells were not activated, IL-7 (1 ng/ml) was added every 2 days. To quantify viral replication, the culture supernatant (100 μl) was taken every 2 days and used for p24 ELISA using an in-house ELISA kit. For HIV-2 infection, cells were similarly pretreated with the actinator, infected for 12 hours, washed, and cultured for 1 week. The culture supernatant (100 μl) was taken every 2 days and used for p27 ELISA using a commercial p27 ELISA kit (XpressBio).

Retroviral particle MSGV-Thy1.1 was assembled using Platinum-E ecotropic packaging cells (Cell BioLabs) cultured in BioCoat 10-cm dishes (Corning). Cells were cotransfected with 18 μg of MSGV-Thy1.1 retroviral vector (Addgene) DNA and 6 μg of pCL-Eco vector (Addgene) DNA using Lipofectamine 2000 (Thermo Fisher Scientific). The viral supernatant was collected after 48 hours. For infection, mouse T cells (1 × 10^6^/ml) were pretreated with B11 (30 μM) for 60 min. The virus and an additional amount of B11 were added to cells for infection for 4 hours. Cells were washed and cultured in fresh medium with recombinant mIL-2 (100 IU/ml), and then activated with anti-CD3/CD28 antibodies for 2 days.

AAV-DJ(GFP) was assembled by cotransfection of HEK293T cells (3 × 10^6^ cells) with pAAV-(GFP), pAAV-DJ, and pHelper (Cell Biolabs) using the EZ-Fectin DNA Transfection kit (Virongy Biosciences) (24 μg of DNA; ratio, 1:1:1). Cells were harvested at 48 hours, and AAV-DJ(GFP) was purified with a Rapid AAV Purification Kit (Virongy Biosciences) without further concentration. CEM-SS CD4 T cells (2 × 10^5^ cell/0.1 ml) were pretreated with B11 for 10 min. AAV-DJ(GFP) (1:100 dilution) and an additional amount of B11 were added to maintain the B11 concentration. Cells were infected for 2 hours. Cells were washed, cultured for 2 days, and then analyzed by flow cytometry for GFP expression.

### Surface and intracellular staining and flow cytometry

For surface staining, single-cell suspensions were prepared in PBS + 0.1% BSA. For CD144, CD324, and CD325 staining, cells were incubated with TruStain FcX Fc blocking buffer (BioLegend) for 30 min at 4°C and then stained with antibodies for 30 min at 4°C. For other surface antibodies, viability dyes, and isotype controls, cells were stained on ice in PBS + 0.1% BSA for 30 min, washed with cold PBS + 0.1% BSA, and then analyzed on a FACSCalibur (BD Biosciences) or LSRFortessa X-20 (BD Biosciences) flow cytometer. For intracellular staining, cells were treated with anti-CD107a for 6 hours in the presence of GolgiPlug (BD Biosciences). Cells were permeabilized and fixed by the Fixation/Permeabilization kit (BD Biosciences) for 20 min at 4°C. Cells were washed once in Perm/Wash buffer (BD Biosciences) and stained with anti–IFN-γ and anti–TNF-α for 40 min at 4°C. Cells were washed with Perm/Wash buffer and analyzed on an LSRFortessa X-20 (BD Biosciences). Antibody information is listed in table S1.

### Cell cycle analysis by 7-amino-actinomycin D and pyronin Y staining

Resting CD4 T cells (1 × 10^6^) were treated with N9 (10 μM) or a scrambled peptide (N20, 10 μM). As a control, cells were also activated with phytohemagglutinin (2 μg/ml) + IL-2 (2 ng/ml). Cells were suspended in 1 ml of 0.03% saponin in PBS and then incubated in 20 μM 7-amino-actinomycin D (Sigma-Aldrich) for 30 min at room temperature in the dark. Cells were kept on ice for 5 min and then incubated with 5 μM pyronin Y (Sigma-Aldrich) for 10 min on ice. Stained cells were directly analyzed by flow cytometry on a FACSCalibur (BD Biosciences).

### Viral entry assays

The BlaM-Vpr–based viral entry assay was performed as previously described (*36*). Briefly, viruses were generated by cotransfection of HEK293T cells with three plasmids: pNL4-3, pAdvantage (Promega), and pCMV4-3BlaM-Vpr (provided by W. C. Greene) (at a ratio of 6:1:2). The supernatant was harvested at 48 hours and concentrated with the Low-Speed Viral Concentration Kit (Virongy Biosciences). Cells (1 × 10^6^) were infected with BlaM-Vpr containing HIV-1(NL4–3) for 4 hours, washed, and then loaded with CCF2 for flow cytometry. Data analysis was performed using FlowJo software (FlowJo).

### Western blot of p-cofilin and HIV proteins

One-half million resting CD4 T cells were treated with N7 (10 μM) for a time course. Cells were pelleted and lysed in NuPAGE LDS Sample Buffer (Thermo Fisher Scientific). Samples were separated by SDS–polyacrylamide gel electrophoresis and transferred onto a nitrocellulose membrane (Thermo Fisher Scientific). The membrane was blocked for 30 min at room temperature with Western Blocking Buffer (Li-Cor Biosciences), washed in TBST [50 mM tris-HCl (pH 7.4), 150 mM NaCl, and 0.1% Tween 20], and incubated overnight at 4°C with a mouse anti-cofilin antibody (1:1000 dilution) (BD Biosciences) and a rabbit anti–p-cofilin (Ser^3^) antibody (1:500 dilution) (Cell Signaling) diluted in 2.5% milk-TBST. The blot was washed and then incubated with goat anti-mouse 680-labeled and goat anti-rabbit 800cw-labeled antibodies (Li-Cor Biosciences) (1:5000 diluted in blocking buffer) for 1 hour at 4°C. The blot was washed and scanned with an Odyssey infrared imager (Li-Cor Biosciences). The blot was also stripped with Restore Plus Western Blot Stripping Buffer (Thermo Fisher Scientific) and reprobed with a goat anti–GAPDH (glyceraldehyde-3-phosphate dehydrogenase) antibody (Abcam) (1:1000 diluted in blocking buffer), followed by washing and incubation with rabbit anti-goat DyLight 800–labeled antibodies (KPL) (1:7500 dilution).

### Chemotaxis assay

For chemotaxis assay, one-half million A3R5.7 CD4 T cells were suspended into 100 μl of RPMI 1640 medium and incubated with N7 (20 μM) for 30 min at 37°C. Cells were added to the upper chamber of a transwell plate (6.5-mm diameter and 5-μm pore size with a polycarbonate membrane) (Corning). The lower chamber was filled with 600 μl of medium premixed with SDF-1 (40 ng/ml) (R&D Systems). The transwell plate was incubated at 37°C for 2 hours, then the upper chamber was removed, and cells in the lower chamber were counted in a Beckman Coulter Z2 cell and particle counter.

### Quantitative real-time PCR amplification

Total cellular DNA was purified using a modified SV total RNA isolation kit, as recommended by the manufacturer (Promega). Quantitative real-time polymerase chain reaction (PCR) analyses of viral late reverse-transcribed DNA were carried out by using the Bio-Rad iQ5 real-time PCR detection system, as described previously (*11*), using the following: forward primer, 5′LTR-U5: 5′-AGATCCCTCAGACCCTTTTAGTCA-3′; reverse primer, 3′ gag: 5′-TTCGCTTTCAAGTCCCTGTTC-3′; the probe, FAM-U5/gag: 5′-(FAM)-TGTGGAAAATCTCTAGCAGTGGCGCC-(BHQ)-3′. Each reaction contained 1× TaqMan Universal PCR Master Mix (Applied Biosystems), 300 nM each of the primers, and 300 nM of the probe. The PCR was carried out at 50°C for 2 min, 95°C for 10 min, and 40 cycles of 95°C for 15 s, and 60°C for 60 s.

### Purification of EVs by differential ultracentrifugation

J1.1 cell culture was grown in RPMI 1640 medium supplemented with EV-depleted FBS (100,000*g* centrifugation for 90 min) for 5 days. Cells were pelleted from media by low-speed centrifugation (500*g*, 10 min). The supernatants were transferred into 50-ml conical tubes (VWR) and centrifuged at 2000*g* for 45 min (2000). The pellets were washed in PBS, combined into one tube, spun down again, and resuspended in 100 to 200 μl of PBS (2000 EVs). The resulting supernatants were transferred into a new set of conical tubes and centrifuged at 10,000*g* for 45 min, and the pellets were resuspended with PBS and combined into a single 1.5-ml tube, yielding the 10,000*g* EVs. The supernatants were transferred to another set of 26.3-ml polycarbonate bottles (Beckman Coulter Inc.) and centrifuged at 100,000*g* for 90 min, and pellets were resuspended with PBS and combined into a single tube (100,000*g* EVs). The supernatant was further centrifuged at 167,000*g* for 4 hours, and the pellets were resuspended with PBS (167,000*g*/4-hour EVs). All isolated EVs were frozen at −80°C for later downstream assays.

### EV labeling by lipophilic fluorescent dye

Isolated EVs were labeled with a lipophilic fluorescence dye—BODIPY 493/503 (Thermo Fisher Scientific). An aliquot of BODIPY (1.5 μl) was mixed with 50 μl of EV, and the mixture was incubated for 30 min at 37°C. After incubation, unincorporated dye was removed using a Sephadex G-10 spin column (bed volume of 0.3 ml) for 2 min at 500*g*, yielding 30 μl of labeled EVs. The concentration of resulting BODIPY-labeled EVs was measured by ZetaView nanoparticle tracking analysis.

### EV uptake analysis using flow cytometry

One million log-phase Jurkat and CCF-STTG1 cells were treated with J1.1 EVs in the presence or absence of B11 (10 μM) at a cell-to-EV ratio of 1:10,000. Cells were treated with B11 for 1 hour in 100 μl of media, and then the volume was brought up to 1 ml. Cells were allowed to incubate for 24 hours at 37°C, pelleted, washed with PBS, and then subjected to flow cytometry. Data analysis was performed using CellQuest (BD Biosciences).

### Preparation of cell lysates and Western blotting for EV uptake analysis

EV-recipient Jurkat cells were pelleted and washed with PBS, resuspended in lysis buffer (50 mM tris-HCl, pH 7.5, 120 mM NaCl, 5 mM EDTA, 0.5% Nonidet P-40, 50 mM NaF, 0.2 mM Na_3_VO_4_, and 1 mM dithiothreitol) and one protease inhibitor cocktail tablet per 50 ml (Roche Applied Science). The cell lysate was incubated on ice for 25 min with vortexing every 5 min. Insoluble cell debris was separated via centrifugation at 20,000*g* at 4°C for 10 min. The protein concentration was measured using the Bradford assay according to the manufacturer’s guideline (Bio-Rad). For Western blot analysis, 15 μl of cell lysate (20 to 30 μg of total protein) was mixed with an equal amount 2× Laemmli Sample Buffer (Bio-Rad) with 10% of 2-mercaptoethanol and incubated at 95°C for 3 min. A total of 30 μl of each sample was loaded onto a 4 to 20% tris/glycine 1.0-mm gel (Thermo Fisher Scientific), separated, and transferred onto polyvinylidene difluoride membranes (Millipore). The membranes were blocked in 5% milk in PBS with 0.1% Tween 20 (PBS-T) for 30 min at 4°C and incubated overnight at 4°C with primary antibodies against the following: HIV-1 gp120 (NIH HIV Reagent Program, cat. no. 522; 1:1000), HIV-1 p24 (NIH HIV Reagent Program, cat. no. 4121, 1:1000), and human actin (Abcam, 1:5000). The membranes were washed three times with PBS-T, incubated with horseradish peroxidase–conjugated secondary goat anti-mouse antibodies (Thermo Fisher Scientific, 1:5000) for 2 hours at 4°C, and then washed twice with PBS-T and once with PBS. The membrane was incubated with a Clarity enhanced chemiluminescence substrate (Bio-Rad), and chemiluminescence was recorded using a ChemiDoc Touch Imaging System (Bio-Rad).

### Confocal fluorescence microscopy

For confocal imaging of actin and surface CXCR4, CD4 T cells (2 × 10^6^ cells) were electroporated with 1 μg of pLifeAct-EGFP using Nucleofactor and Nucleofector Kit R (Lonza). At 48 hours postelectroporation, cells were stained with phycoerythrin-labeled anti-CXCR4 antibodies and kept on ice for confocal imaging. Cells were imaged using a Carl Zeiss Laser Scanning Microscope Zen 780 (Carl Zeiss, Thornwood, NY) with a Plan-Apochromat 60×/1.4 Oil DIC M27 objective. Imaging data were exported as .tif files using Zen software and further processed using Adobe Photoshop.

## References

[1] M. Elzinga, J. H. Collins, W. M. Kuehl, R. S. Adelstein, Complete amino-acid sequence of actin of rabbit skeletal muscle. Proc. Natl. Acad. Sci. U.S.A. 70, 2687–2691 (1973). 4517681 10.1073/pnas.70.9.2687 PMC427084

[2] K. C. Holmes, D. Popp, W. Gebhard, W. Kabsch, Atomic model of the actin filament. Nature 347, 44–49 (1990). 2395461 10.1038/347044a0

[3] W. Kabsch, H. G. Mannherz, D. Suck, E. F. Pai, K. C. Holmes, Atomic structure of the actin: DNase I complex. Nature 347, 37–44 (1990). 2395459 10.1038/347037a0

[4] C. G. dos Remedios, D. Chhabra, M. Kekic, I. V. Dedova, M. Tsubakihara, D. A. Berry, N. J. Nosworthy, Actin binding proteins: Regulation of cytoskeletal microfilaments. Physiol. Rev. 83, 433–473 (2003). 12663865 10.1152/physrev.00026.2002

[5] B. Bugyi, M. Kellermayer, The discovery of actin: “To see what everyone else has seen, and to think what nobody has thought”. J. Muscle Res. Cell Motil. 41, 3–9 (2020). 31093826 10.1007/s10974-019-09515-z PMC7109165

[6] L. Dupré, R. Houmadi, C. Tang, J. Rey-Barroso, T lymphocyte migration: An action movie starring the actin and associated actors. Front. Immunol. 6, 586 (2015). 26635800 10.3389/fimmu.2015.00586 PMC4649030

[7] T. J. Mitchison, L. P. Cramer, Actin-based cell motility and cell locomotion. Cell 84, 371–379 (1996). 8608590 10.1016/s0092-8674(00)81281-7

[8] C. L. Adams, W. J. Nelson, S. J. Smith, Quantitative analysis of cadherin-catenin-actin reorganization during development of cell-cell adhesion. J. Cell Biol. 135, 1899–1911 (1996). 8991100 10.1083/jcb.135.6.1899 PMC2133977

[9] P. K. Mattila, F. D. Batista, B. Treanor, Dynamics of the actin cytoskeleton mediates receptor cross talk: An emerging concept in tuning receptor signaling. J. Cell Biol. 212, 267–280 (2016). 26833785 10.1083/jcb.201504137 PMC4748574

[10] B. Qualmann, M. M. Kessels, R. B. Kelly, Molecular links between endocytosis and the actin cytoskeleton. J. Cell Biol. 150, F111–F116 (2000). 10974009 10.1083/jcb.150.5.f111 PMC2175242

[11] A. Yoder, D. Yu, L. Dong, S. R. Iyer, X. Xu, J. Kelly, J. Liu, W. Wang, P. J. Vorster, L. Agulto, D. A. Stephany, J. N. Cooper, J. W. Marsh, Y. Wu, HIV envelope-CXCR4 signaling activates cofilin to overcome cortical actin restriction in resting CD4 T cells. Cell 134, 782–792 (2008). 18775311 10.1016/j.cell.2008.06.036 PMC2559857

[12] M. Spear, Y. Wu, Viral exploitation of actin: Force-generation and scaffolding functions in viral infection. Virol. Sin. 29, 139–147 (2014). 24938714 10.1007/s12250-014-3476-0 PMC4373664

[13] P. J. Vorster, J. Guo, A. Yoder, W. Wang, Y. Zheng, X. Xu, D. Yu, M. Spear, Y. Wu, LIM kinase 1 modulates cortical actin and CXCR4 cycling and is activated by HIV-1 to initiate viral infection. J. Biol. Chem. 286, 12554–12564 (2011). 21321123 10.1074/jbc.M110.182238 PMC3069457

[14] M. Spear, J. Guo, A. Turner, D. Yu, W. Wang, B. Meltzer, S. He, X. Hu, H. Shang, J. Kuhn, Y. Wu, HIV-1 triggers WAVE2 phosphorylation in primary CD4 T cells and macrophages, mediating Arp2/3-dependent nuclear migration. J. Biol. Chem. 289, 6949–6959 (2014). 24415754 10.1074/jbc.M113.492132 PMC3945356

[15] W. Wang, J. Guo, D. Yu, P. J. Vorster, W. Chen, Y. Wu, A dichotomy in cortical actin and chemotactic actin activity between human memory and naive T cells contributes to their differential susceptibility to HIV-1 infection. J. Biol. Chem. 287, 35455–35469 (2012). 22879601 10.1074/jbc.M112.362400 PMC3471682

[16] F. Yi, J. Guo, D. Dabbagh, M. Spear, S. He, K. Kehn-Hall, J. Fontenot, Y. Yin, M. Bibian, C. M. Park, K. Zheng, H. Park, V. Soloveva, D. Gharaibeh, C. Retterer, R. Zamani, M. L. Pitt, J. Naughton, Y. Jiang, H. Shang, R. M. Hakami, B. Ling, J. A. Young, S. Bavari, X. Xu, Y. Feng, Y. Wu, Discovery of novel small molecule inhibitors of LIM domain kinase for inhibiting HIV-1. J. Virol. 91, e02418-16 (2017). 28381571 10.1128/JVI.02418-16 PMC5469273

[17] J. Guo, W. Wang, D. Yu, Y. Wu, Spinoculation triggers dynamic actin and cofilin activity facilitating HIV-1 infection of transformed and resting CD4 T cells. J. Virol. 85, 9824–9833 (2011). 21795326 10.1128/JVI.05170-11 PMC3196392

[18] Y. Xiang, K. Zheng, H. Ju, S. Wang, Y. Pei, W. Ding, Z. Chen, Q. Wang, X. Qiu, M. Zhong, F. Zeng, Z. Ren, C. Qian, G. Liu, K. Kitazato, Y. Wang, Cofilin 1-mediated biphasic F-actin dynamics of neuronal cells affect herpes simplex virus 1 infection and replication. J. Virol. 86, 8440–8451 (2012). 22623803 10.1128/JVI.00609-12 PMC3421760

[19] N. Müller, E. Avota, J. Schneider-Schaulies, H. Harms, G. Krohne, S. Schneider-Schaulies, Measles virus contact with T cells impedes cytoskeletal remodeling associated with spreading, polarization, and CD3 clustering. Traffic 7, 849–858 (2006). 16787397 10.1111/j.1600-0854.2006.00426.x

[20] R. Koga, Y. Sugita, T. Noda, Y. Yanagi, S. Ohno, Actin-modulating protein cofilin is involved in the formation of measles virus ribonucleoprotein complex at the perinuclear region. J. Virol. 89, 10524–10531 (2015). 26269174 10.1128/JVI.01819-15 PMC4580163

[21] T. Jacob, C. Van den Broeke, M. van Troys, D. Waterschoot, C. Ampe, H. W. Favoreel, Alphaherpesviral US3 kinase induces cofilin dephosphorylation to reorganize the actin cytoskeleton. J. Virol. 87, 4121–4126 (2013). 23365433 10.1128/JVI.03107-12 PMC3624233

[22] Y. Nie, L. Hui, M. Guo, W. Yang, R. Huang, J. Chen, X. Wen, M. Zhao, Y. Wu, Rearrangement of actin cytoskeleton by zika virus infection facilitates blood-testis barrier hyperpermeability. Virol. Sin. 36, 692–705 (2021). 33534087 10.1007/s12250-020-00343-x PMC8379325

[23] H. Wang, Y. Liu, W. Liu, K. Wu, X. Wang, F-actin dynamics in midgut cells enables virus persistence in vector insects. Mol. Plant Pathol. 23, 1671–1685 (2022). 36073369 10.1111/mpp.13260 PMC9562576

[24] M. Spear, J. Guo, Y. Wu, Novel anti-HIV therapeutics targeting chemokine receptors and actin regulatory pathways. Immunol. Rev. 256, 300–312 (2013). 24117829 10.1111/imr.12106 PMC3888822

[25] M. Spear, J. Guo, Y. Wu, The trinity of the cortical actin in the initiation of HIV-1 infection. Retrovirology 9, 45 (2012). 22640593 10.1186/1742-4690-9-45 PMC3416652

[26] M. G. Lana, B. E. Strauss, Production of lentivirus for the establishment of CAR-T cells. Methods Mol. Biol. 2086, 61–67 (2020). 31707667 10.1007/978-1-0716-0146-4_4

[27] M. D. Welch, D. A. Holtzman, D. G. Drubin, The yeast actin cytoskeleton. Curr. Opin. Cell Biol. 6, 110–119 (1994). 8167016 10.1016/0955-0674(94)90124-4

[28] L. R. Otterbein, P. Graceffa, R. Dominguez, The crystal structure of uncomplexed actin in the ADP state. Science 293, 708–711 (2001). 11474115 10.1126/science.1059700

[29] R. Dominguez, Actin-binding proteins—A unifying hypothesis. Trends Biochem. Sci. 29, 572–578 (2004). 15501675 10.1016/j.tibs.2004.09.004

[30] R. Dominguez, K. C. Holmes, Actin structure and function. Annu. Rev. Biophys. 40, 169–186 (2011). 21314430 10.1146/annurev-biophys-042910-155359 PMC3130349

[31] T. D. Pollard, Actin and actin-binding proteins. Cold Spring Harb. Perspect. Biol. 8, a018226 (2016). 26988969 10.1101/cshperspect.a018226 PMC4968159

[32] E. Vivès, P. Brodin, B. Lebleu, A truncated HIV-1 Tat protein basic domain rapidly translocates through the plasma membrane and accumulates in the cell nucleus. J. Biol. Chem. 272, 16010–16017 (1997). 9188504 10.1074/jbc.272.25.16010

[33] F. Milletti, Cell-penetrating peptides: Classes, origin, and current landscape. Drug Discov. Today 17, 850–860 (2012). 22465171 10.1016/j.drudis.2012.03.002

[34] Y. Wu, M. H. Beddall, J. W. Marsh, Rev-dependent indicator T cell line. Curr. HIV Res. 5, 395–403 (2007). 10.2174/157016207781024018 PMC2366165 17627502

[35] D. Unutmaz, V. N. KewalRamani, S. Marmon, D. R. Littman, Cytokine signals are sufficient for HIV-1 infection of resting human T lymphocytes. J. Exp. Med. 189, 1735–1746 (1999). 10359577 10.1084/jem.189.11.1735 PMC2193071

[36] M. Cavrois, C. De Noronha, W. C. Greene, A sensitive and specific enzyme-based assay detecting HIV-1 virion fusion in primary T lymphocytes. Nat. Biotechnol. 20, 1151–1154 (2002). 12355096 10.1038/nbt745

[37] R. Kalluri, V. S. LeBleu, The biology, function, and biomedical applications of exosomes. Science 367, eaau6977 (2020). 32029601 10.1126/science.aau6977 PMC7717626

[38] S. L. N. Maas, X. O. Breakefield, A. M. Weaver, Extracellular vesicles: Unique intercellular delivery vehicles. Trends Cell Biol. 27, 172–188 (2017). 27979573 10.1016/j.tcb.2016.11.003 PMC5318253

[39] I. K. Herrmann, M. J. A. Wood, G. Fuhrmann, Extracellular vesicles as a next-generation drug delivery platform. Nat. Nanotechnol. 16, 748–759 (2021). 34211166 10.1038/s41565-021-00931-2

[40] Y. Kim, G. A. Mensah, S. Al Sharif, D. O. Pinto, H. Branscome, S. V. Yelamanchili, M. Cowen, J. Erickson, P. Khatkar, R. Mahieux, F. Kashanchi, Extracellular vesicles from infected cells are released prior to virion release. Cells 10, 781 (2021). 33916140 10.3390/cells10040781 PMC8066806

[41] J. R. Bamburg, Proteins of the ADF/cofilin family: Essential regulators of actin dynamics. Annu. Rev. Cell Dev. Biol. 15, 185–230 (1999). 10611961 10.1146/annurev.cellbio.15.1.185

[42] W. Humphrey, A. Dalke, K. Schulten, VMD: Visual molecular dynamics. J. Mol. Graph. 14, 33-38, 27–38 (1996). 10.1016/0263-7855(96)00018-5 8744570

[43] N. Yang, O. Higuchi, K. Ohashi, K. Nagata, A. Wada, K. Kangawa, E. Nishida, K. Mizuno, Cofilin phosphorylation by LIM-kinase 1 and its role in Rac-mediated actin reorganization. Nature 393, 809–812 (1998). 9655398 10.1038/31735

[44] G. D. Rose, A. R. Geselowitz, G. J. Lesser, R. H. Lee, M. H. Zehfus, Hydrophobicity of amino acid residues in globular proteins. Science 229, 834–838 (1985). 4023714 10.1126/science.4023714

[45] B. W. Bernstein, J. R. Bamburg, ADF/cofilin: A functional node in cell biology. Trends Cell Biol. 20, 187–195 (2010). 20133134 10.1016/j.tcb.2010.01.001 PMC2849908

[46] T. M. Svitkina, G. G. Borisy, Arp2/3 complex and actin depolymerizing factor/cofilin in dendritic organization and treadmilling of actin filament array in lamellipodia. J. Cell Biol. 145, 1009–1026 (1999). 10352018 10.1083/jcb.145.5.1009 PMC2133125

[47] M. R. Bubb, I. Spector, B. B. Beyer, K. M. Fosen, Effects of jasplakinolide on the kinetics of actin polymerization. An explanation for certain in vivo observations. J. Biol. Chem. 275, 5163–5170 (2000). 10671562 10.1074/jbc.275.7.5163

[48] H. G. Mannherz, E. Ballweber, M. Galla, S. Villard, C. Granier, C. Steegborn, A. Schmidtmann, K. Jaquet, B. Pope, A. G. Weeds, Mapping the ADF/cofilin binding site on monomeric actin by competitive cross-linking and peptide array: Evidence for a second binding site on monomeric actin. J. Mol. Biol. 366, 745–755 (2007). 17196218 10.1016/j.jmb.2006.11.100

[49] J. K. A. Kamal, S. A. Benchaar, K. Takamoto, E. Reisler, M. R. Chance, Three-dimensional structure of cofilin bound to monomeric actin derived by structural mass spectrometry data. Proc. Natl. Acad. Sci. U.S.A. 104, 7910–7915 (2007). 17470807 10.1073/pnas.0611283104 PMC1876546

[50] K. Hori, F. Morita, Actin-actin contact: Inhibition of actin-polymerization by subdomain 4 peptide fragments. J. Biochem. 112, 401–408 (1992). 1429530 10.1093/oxfordjournals.jbchem.a123912

[51] K. Tanaka, S. Takeda, K. Mitsuoka, T. Oda, C. Kimura-Sakiyama, Y. Maéda, A. Narita, Structural basis for cofilin binding and actin filament disassembly. Nat. Commun. 9, 1860 (2018). 29749375 10.1038/s41467-018-04290-w PMC5945598

[52] L. Blanchoin, T. D. Pollard, R. D. Mullins, Interactions of ADF/cofilin, Arp2/3 complex, capping protein and profilin in remodeling of branched actin filament networks. Curr. Biol. 10, 1273–1282 (2000). 11069108 10.1016/s0960-9822(00)00749-1

[53] X. Xu, S. Bhimani, H. Pots, X. Wen, T. J. Jeon, A. Kortholt, T. Jin, Membrane targeting of C2GAP1 enables *Dictyostelium discoideum* to sense chemoattractant gradient at a higher concentration range. Front. Cell Dev. Biol. 9, 725073 (2021). 34395450 10.3389/fcell.2021.725073 PMC8362602

[54] X. Xu, X. Wen, A. Moosa, S. Bhimani, T. Jin, Ras inhibitor CAPRI enables neutrophil-like cells to chemotax through a higher-concentration range of gradients. Proc. Natl. Acad. Sci. U.S.A. 118, e2002162118 (2021). 34675073 10.1073/pnas.2002162118 PMC8639426

[55] X. Xu, X. Wen, D. M. Veltman, I. Keizer-Gunnink, H. Pots, A. Kortholt, T. Jin, GPCR-controlled membrane recruitment of negative regulator C2GAP1 locally inhibits Ras signaling for adaptation and long-range chemotaxis. Proc. Natl. Acad. Sci. U.S.A. 114, E10092–E10101 (2017). 29109256 10.1073/pnas.1703208114 PMC5703269

[56] G. Bloomfield, D. Traynor, S. P. Sander, D. M. Veltman, J. A. Pachebat, R. R. Kay, Neurofibromin controls macropinocytosis and phagocytosis in *Dictyostelium*. eLife 4, e04940 (2015). 25815683 10.7554/eLife.04940 PMC4374526

[57] D. M. Veltman, M. G. Lemieux, D. A. Knecht, R. H. Insall, PIP_3_-dependent macropinocytosis is incompatible with chemotaxis. J. Cell Biol. 204, 497–505 (2014). 24535823 10.1083/jcb.201309081 PMC3926956

[58] D. M. Veltman, T. D. Williams, G. Bloomfield, B.-C. Chen, E. Betzig, R. H. Insall, R. R. Kay, A plasma membrane template for macropinocytic cups. eLife 5, e20085 (2016). 27960076 10.7554/eLife.20085 PMC5154761

[59] X. Xu, H. Pots, B. K. Gilsbach, D. Parsons, D. M. Veltman, S. G. Ramachandra, H. Li, A. Kortholt, T. Jin, C2GAP2 is a common regulator of Ras signaling for chemotaxis, phagocytosis, and macropinocytosis. Front. Immunol. 13, 1075386 (2022). 36524124 10.3389/fimmu.2022.1075386 PMC9745196

[60] B. S. Stein, S. D. Gowda, J. D. Lifson, R. C. Penhallow, K. G. Bensch, E. G. Engleman, pH-independent HIV entry into CD4-positive T cells via virus envelope fusion to the plasma membrane. Cell 49, 659–668 (1987). 3107838 10.1016/0092-8674(87)90542-3

[61] D. Yu, W. Wang, A. Yoder, M. Spear, Y. Wu, The HIV envelope but not VSV glycoprotein is capable of mediating HIV latent infection of resting CD4 T cells. PLOS Pathog. 5, e1000633 (2009). 19851458 10.1371/journal.ppat.1000633 PMC2760144

[62] S. Gurung, D. Perocheau, L. Touramanidou, J. Baruteau, The exosome journey: From biogenesis to uptake and intracellular signalling. Cell Commun. Signal. 19, 47 (2021). 33892745 10.1186/s12964-021-00730-1 PMC8063428

[63] B. J. Galletta, J. A. Cooper, Actin and endocytosis: Mechanisms and phylogeny. Curr. Opin. Cell Biol. 21, 20–27 (2009). 19186047 10.1016/j.ceb.2009.01.006 PMC2670849

[64] A. I. Bachir, A. R. Horwitz, W. J. Nelson, J. M. Bianchini, Actin-based adhesion modules mediate cell interactions with the extracellular matrix and neighboring cells. Cold Spring Harb. Perspect. Biol. 9, a023234 (2017). 28679638 10.1101/cshperspect.a023234 PMC5495059

[65] C. M. Nelson, D. M. Pirone, J. L. Tan, C. S. Chen, Vascular endothelial-cadherin regulates cytoskeletal tension, cell spreading, and focal adhesions by stimulating RhoA. Mol. Biol. Cell 15, 2943–2953 (2004). 15075376 10.1091/mbc.E03-10-0745 PMC420116

[66] L. Labanieh, C. L. Mackall, CAR immune cells: Design principles, resistance and the next generation. Nature 614, 635–648 (2023). 36813894 10.1038/s41586-023-05707-3

[67] F. Müller, J. Taubmann, L. Bucci, A. Wilhelm, C. Bergmann, S. Völkl, M. Aigner, T. Rothe, I. Minopoulou, C. Tur, J. Knitza, S. Kharboutli, S. Kretschmann, I. Vasova, S. Spoerl, H. Reimann, L. Munoz, R. G. Gerlach, S. Schäfer, R. Grieshaber-Bouyer, A. S. Korganow, D. Farge-Bancel, D. Mougiakakos, A. Bozec, T. Winkler, G. Krönke, A. Mackensen, G. Schett, CD19 CAR T-cell therapy in autoimmune disease—A case series with follow-up. N. Engl. J. Med. 390, 687–700 (2024). 38381673 10.1056/NEJMoa2308917

[68] M. Ayala Ceja, M. Khericha, C. M. Harris, C. Puig-Saus, Y. Y. Chen, CAR-T cell manufacturing: Major process parameters and next-generation strategies. J. Exp. Med. 221, e20230903 (2024). 38226974 10.1084/jem.20230903 PMC10791545

[69] A. P. Cribbs, A. Kennedy, B. Gregory, F. M. Brennan, Simplified production and concentration of lentiviral vectors to achieve high transduction in primary human T cells. BMC Biotechnol. 13, 98 (2013). 24215295 10.1186/1472-6750-13-98 PMC3830501

[70] D. S. Ory, B. A. Neugeboren, R. C. Mulligan, A stable human-derived packaging cell line for production of high titer retrovirus/vesicular stomatitis virus G pseudotypes. Proc. Natl. Acad. Sci. U.S.A. 93, 11400–11406 (1996). 8876147 10.1073/pnas.93.21.11400 PMC38069

[71] L. M. Agosto, J. J. Yu, M. K. Liszewski, C. Baytop, N. Korokhov, L. M. Humeau, U. O’Doherty, The CXCR4-tropic human immunodeficiency virus envelope promotes more-efficient gene delivery to resting CD4^+^ T cells than the vesicular stomatitis virus glycoprotein G envelope. J. Virol. 83, 8153–8162 (2009). 19493998 10.1128/JVI.00220-09 PMC2715791

[72] T. L. A. Wachsmann, A. K. Wouters, D. F. G. Remst, R. S. Hagedoorn, M. H. Meeuwsen, E. van Diest, J. Leusen, J. Kuball, J. H. F. Falkenburg, M. H. M. Heemskerk, Comparing CAR and TCR engineered T cell performance as a function of tumor cell exposure. Oncoimmunology 11, 2033528 (2022). 35127255 10.1080/2162402X.2022.2033528 PMC8812760

[73] S. Ghassemi, S. Nunez-Cruz, R. S. O’Connor, J. A. Fraietta, P. R. Patel, J. Scholler, D. M. Barrett, S. M. Lundh, M. M. Davis, F. Bedoya, C. Zhang, J. Leferovich, S. F. Lacey, B. L. Levine, S. A. Grupp, C. H. June, J. J. Melenhorst, M. C. Milone, Reducing ex vivo culture improves the antileukemic activity of chimeric antigen receptor (CAR) T cells. Cancer Immunol. Res. 6, 1100–1109 (2018). 30030295 10.1158/2326-6066.CIR-17-0405 PMC8274631

[74] S. Ghassemi, J. S. Durgin, S. Nunez-Cruz, J. Patel, J. Leferovich, M. Pinzone, F. Shen, K. D. Cummins, G. Plesa, V. A. Cantu, S. Reddy, F. D. Bushman, S. I. Gill, U. O’Doherty, R. S. O’Connor, M. C. Milone, Rapid manufacturing of non-activated potent CAR T cells. Nat. Biomed. Eng. 6, 118–128 (2022). 35190680 10.1038/s41551-021-00842-6 PMC8860360

[75] T. Huan, D. Chen, G. Liu, H. Zhang, X. Wang, Z. Wu, Y. Wu, Q. Xu, F. Yu, Activation-induced cell death in CAR-T cell therapy. Hum. Cell 35, 441–447 (2022). 35032297 10.1007/s13577-022-00670-z

[76] R. C. Sterner, R. M. Sterner, CAR-T cell therapy: Current limitations and potential strategies. Blood Cancer J. 11, 69 (2021). 33824268 10.1038/s41408-021-00459-7 PMC8024391

[77] T. L. Hunter, Y. Bao, Y. Zhang, D. Matsuda, R. Riener, A. Wang, J. J. Li, F. Soldevila, D. S. H. Chu, D. P. Nguyen, Q. C. Yong, B. Ross, M. Nguyen, J. Vestal, S. Roberts, D. Galvan, J. B. Vega, D. Jhung, M. Butcher, J. Nguyen, S. Zhang, C. Fernandez, J. Chen, C. Herrera, Y. Kuo, E. M. Pica, G. Mondal, A. L. Mammen, J. Scholler, S. P. Tanis, S. A. Sievers, A. M. Frantz, G. B. Adams, L. Shawver, R. Farzaneh-Far, M. Rosenzweig, P. P. Karmali, A. I. Bot, C. H. June, H. Aghajanian, In vivo CAR T cell generation to treat cancer and autoimmune disease. Science 388, 1311–1317 (2025). 40536974 10.1126/science.ads8473

[78] J. I. Andorko, R. M. Russell, B. C. Schnepp, D. Grubaugh, K. F. Mullen, A. Wakabayashi, L. J. Carrington, T. O’Malley, L. Kuri-Cervantes, T. D. Culp, P. R. Johnson, Targeted in vivo delivery of genetic medicines utilizing an engineered lentiviral vector platform results in CAR T and NK cell generation. Mol. Ther. 33, 4937–4952 (2025). 40581818 10.1016/j.ymthe.2025.06.036 PMC12848171

[79] T. Coradin, A. L. Keating, A. R. Barnard, L. Whilding, D. Pombal, Z. Hannoun, J. Lewis, G. Devarajan, S. Iqball, E. Burton, S. Ferluga, D. M. Jones, B. M. Alberts, J. Wright, D. C. Farley, D. M. O’Connor, R. M. Rao, K. A. Mitrophanous, Y. Lad, R. Nimmo, Efficient in vivo generation of CAR T cells using a retargeted fourth-generation lentiviral vector. Mol. Ther. 33, 4953–4967 (2025). 40676833 10.1016/j.ymthe.2025.07.006 PMC12848154

[80] Y. Wu, M. H. Beddall, J. W. Marsh, Rev-dependent lentiviral expression vector. Retrovirology 4, 12 (2007). 17286866 10.1186/1742-4690-4-12 PMC1797186

