# Supplementary material for: Discovery of actinators, actin-derived bioactive peptides that modulate cytoskeleton and actin-related cellular activities
Source: Sci Adv. 2026 Apr 17;12(16):eaeb5548. doi: 10.1126/sciadv.aeb5548 (PMC13089335; doi:10.1126/sciadv.aeb5548)
Supplement: Supplementary file 1 — Figs. S1 to S15 Table S1 [file sciadv.aeb5548_sm.pdf]

Science Advances
AAAS

Supplementary Materials for

Discovery of actinators, actin-derived bioactive peptides that modulate cytoskeleton and actin-related cellular activities

Fei Yi *et al.*

Corresponding author: Yuntao Wu, ywu8@gmu.edu

Sci. Adv. **12**, eaeb5548 (2026)
DOI: 10.1126/sciadv.aeb5548

This PDF file includes:

Figs. S1 to S15
Table S1

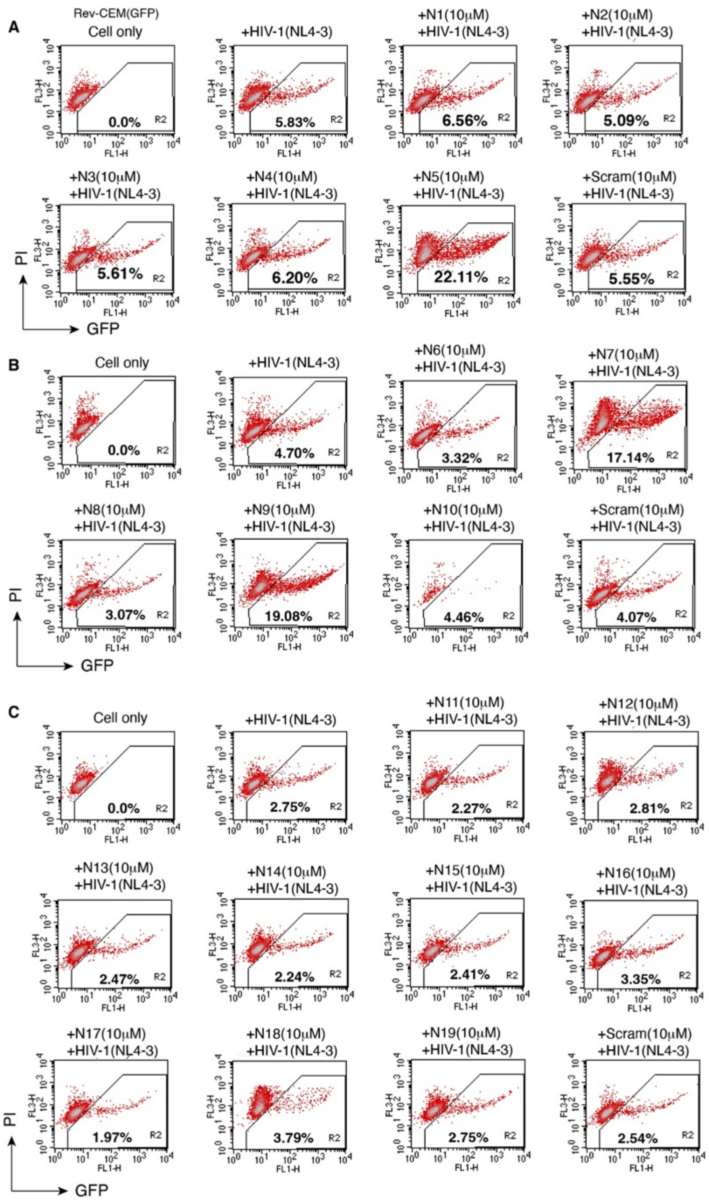

Fig. S1.

Screening for bioactive peptides using HIV-1 infection of Rev-dependent indicator cells. (A to C) Rev-CEM-GFP cells were pre-treated with 10 μM of each peptide for 1 hour, infected with HIV-1(NL4-3) for 2 hours, washed with medium, and then cultured for 2 days. HIV-1 replication was quantified by GFP expression. Propidium iodide (PI) was used during flow cytometry to quantify HIV-1 replication only in live cells. As a control, an actin sequence scrambled peptide (Scram) was used.

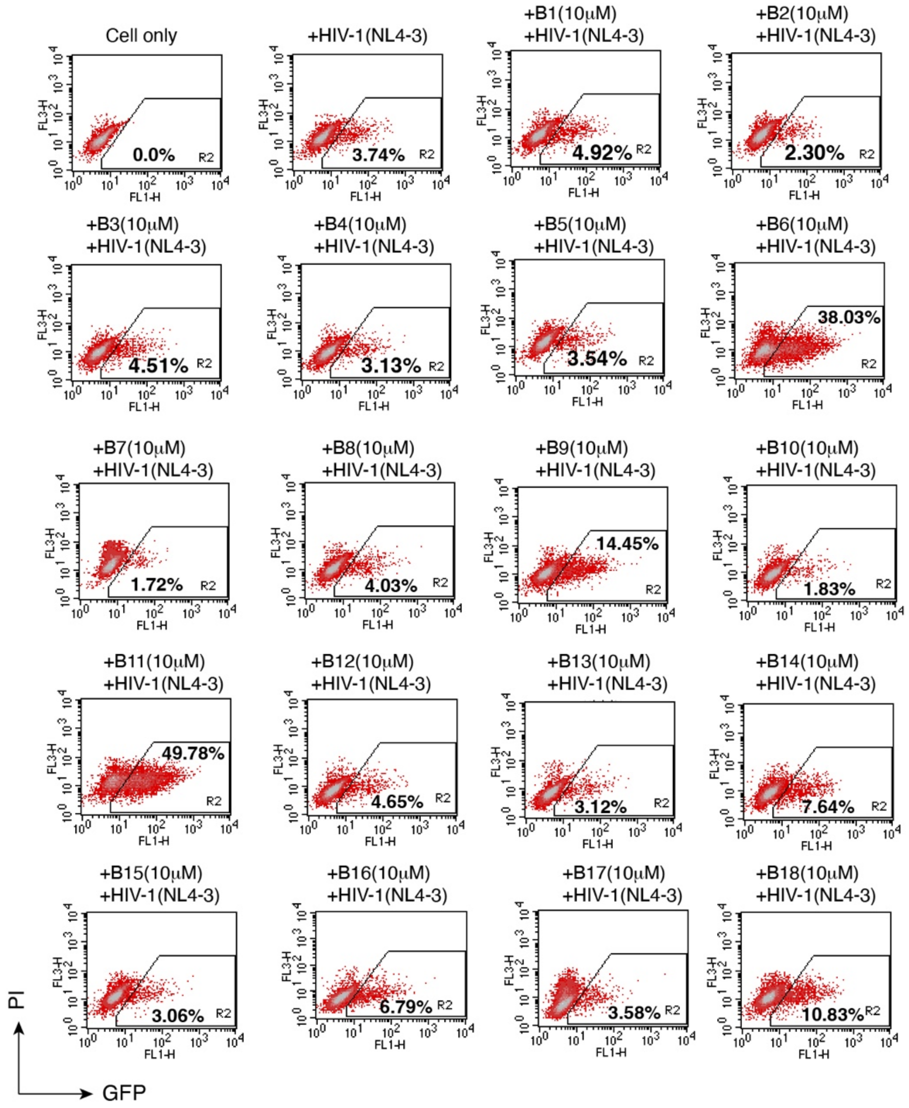

Fig. S2.

Screening for bioactive peptides using HIV-1 infection of Rev-dependent indicator cells. Rev-CEM-GFP/Luc indicator cells were pre-treated with 10 μM of each peptide for 1 hour, infected with HIV-1(NL4-3) for 2 hours, washed with medium, and then cultured for 2 days. HIV-1 replication was quantified by GFP expression. Propidium iodide (PI) was used during flow cytometry to quantify HIV-1 replication only in live cells.

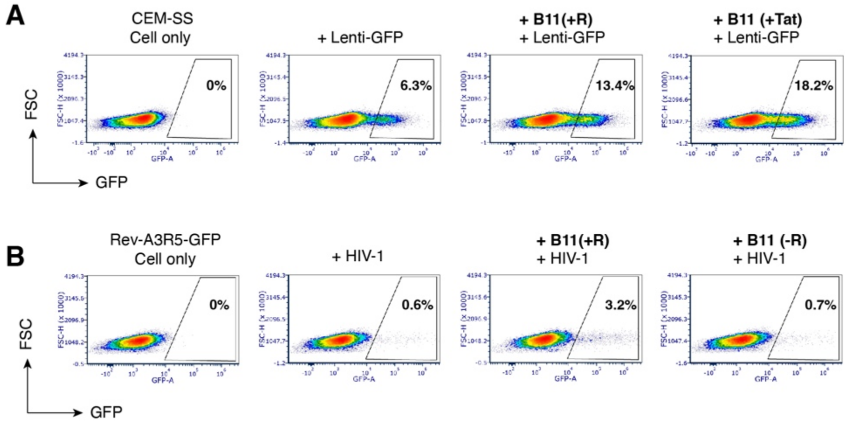

Fig. S3.

The requirement of cell-penetrating peptides for the bioactivity of actinator B11. (A) The cell-penetrating peptides, polyarginine (+R) and HIV Tat-peptide (+Tat), are connected to the actin peptide B11 during peptide synthesis. The peptides were used to enhance lentiviral vector (Lenti-GFP) transduction of human CD4 T cells, CEM-SS. In the experiments, cells were pre-treated with B11(+R) or B11(+Tat) (10 μM) for 30 minutes, and then infected with Lent-GFP viral particles for 2 hours. Following infection, cells were washed and cultured for 2 days. GFP expression was quantified by flow cytometry. (B) The requirement of cell-penetrating peptides for actinator-mediated enhancement of viral infection. Rev-A3R5-GFP indicator CD4 T cells were pre-treated with B11(+R) or B11(-R) (30 μM) and then infected with HIV-1 for 2 hours. Following infection, cells were washed and cultured for 2 days. GFP expression was quantified by flow cytometry. For B11(-R), the polyarginine peptide was removed from B11 during peptide synthesis.

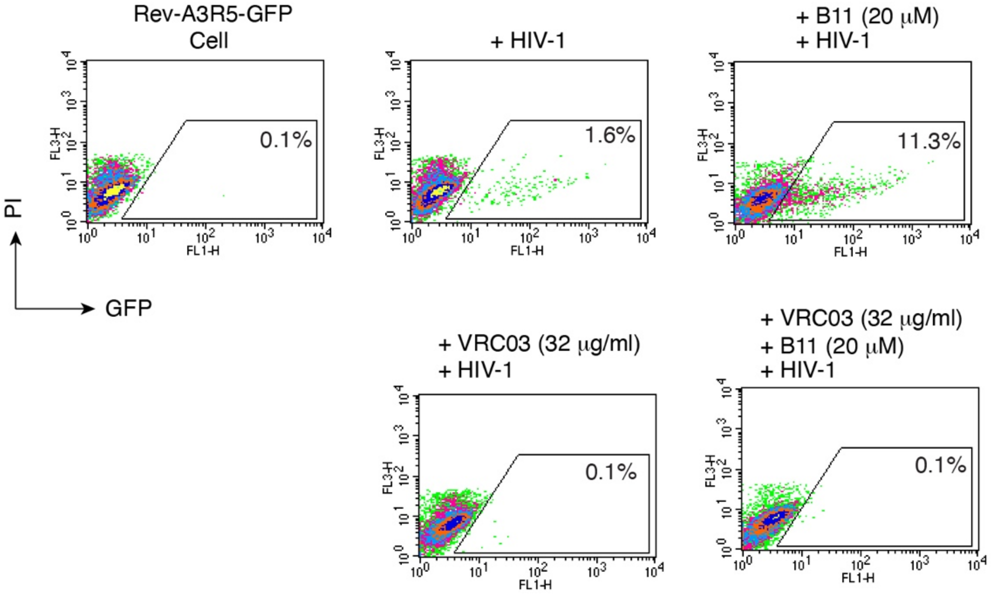

Fig. S4.

Inhibition of B11-mediated enhancement of HIV-1 infection by VRC03. Rev-A3R5-GFP T cells were not pre-treated or pre-treated with B11 (20 μM) for 60 minutes, and then infected with HIV-1 for 3 hours. In the lower panel, HIV-1 particles were also pre-incubated with a neutralizing antibody VRC03 for 1 hour and then added to cells for infection for 3 hours in the presence or absence of B11 treatment. Cells were washed and cultured for 2 days. HIV-1 replication was quantified by GFP expression. Propidium iodide (PI) was used during flow cytometry to quantify HIV-1 replication only in live cells.

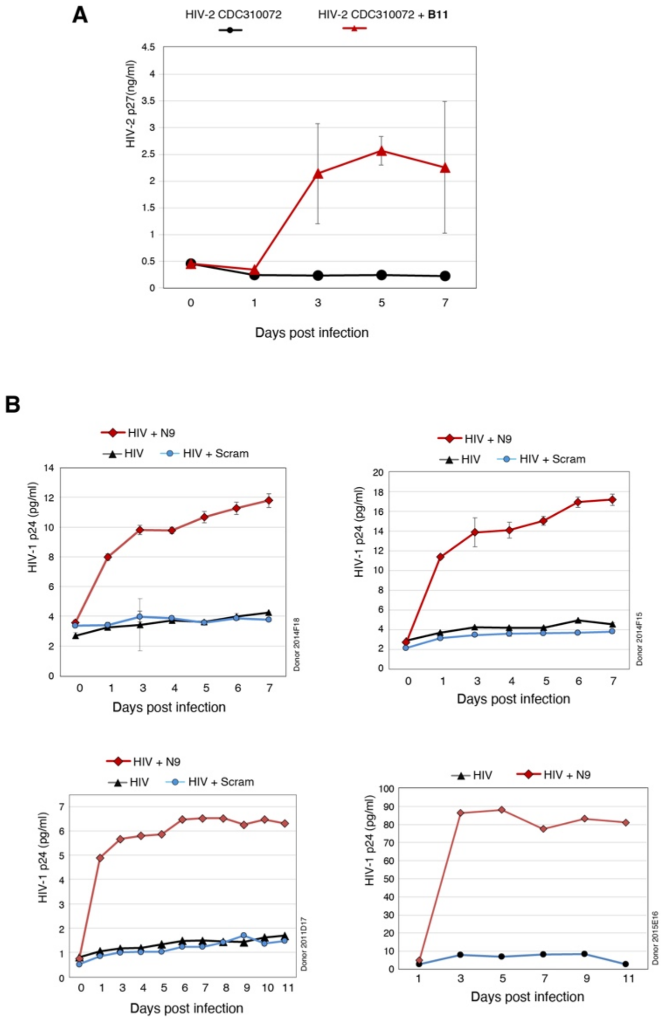

Fig. S5.

Actinator B11 and N9 enhance HIV-1 and HIV-2 infection. (A) Actinator B11 enhances HIV-2(CDC310072) infection of A3R5.7 CD4 T cells. Cells were pre-treated with B11 (10 μM) for 10 minutes and then infected with HIV-2(CDC310072) for 12 hours. Cells were washed and cultured in medium for 7 days. HIV-2 replication was quantified by p27 ELISA. (B) Actinator N9 enhances HIV-1 latent infection of resting CD4 T cells. Cells were pre-treated with N9 (10 μM) or a scrambled peptide (Scram, 10 μM), infected with HIV-1(NL4-3) for 2 hours, washed, and then cultured with IL-7 (10 ng/ml) without T cell activation for 7 days. HIV-1 replication was quantified by p24 ELISA.

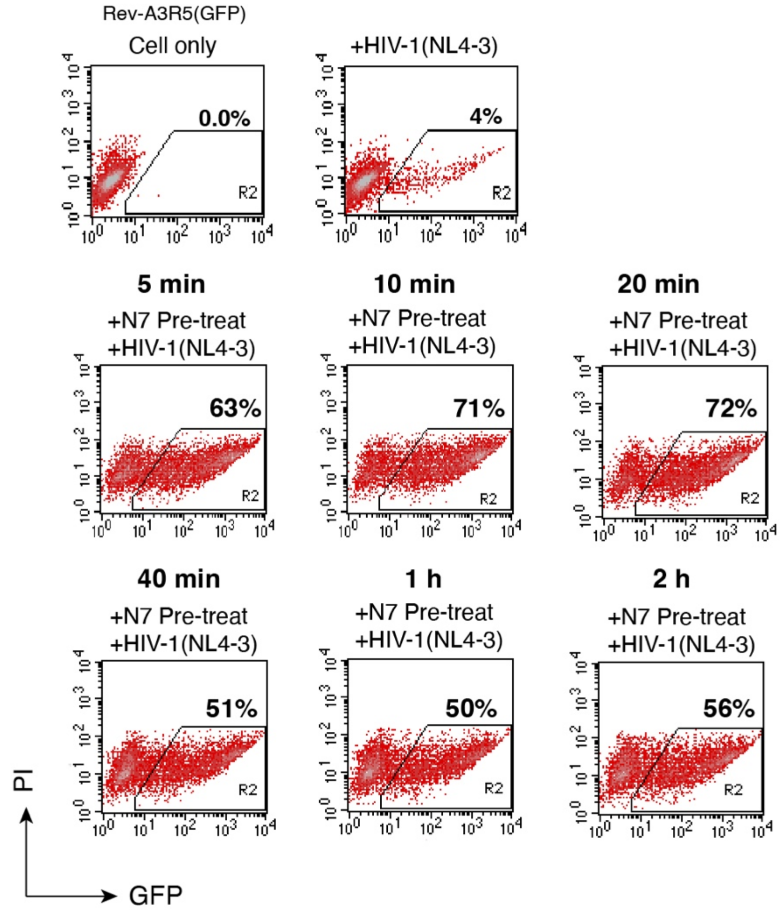

Fig. S6.

Actinator enhances HIV-1 infection can occur rapidly after a brief treatment of cells before infection. Rev-A3R5(GFP) cells were pre-treated with 10 μM of N7 from 5 minutes to 2 hours, infected with HIV-1(NL4-3) for 2 hours, washed with medium, and then cultured for 2 days. HIV-1 replication was quantified by GFP expression. Propidium iodide (PI) was used during flow cytometry to quantify HIV-1 replication only in live cells.

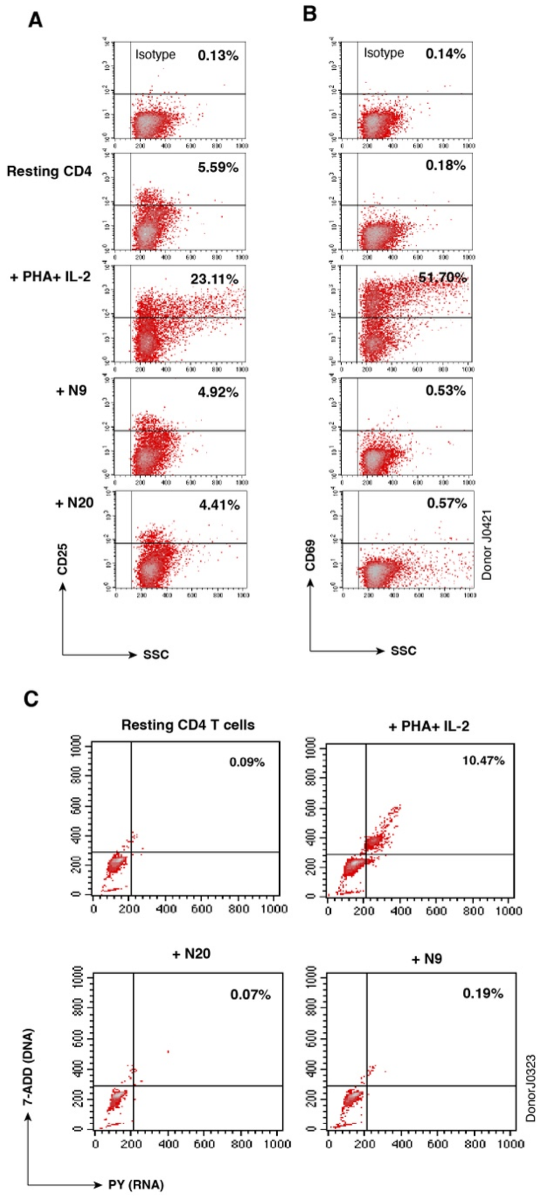

Fig. S7.

Actinator N9 does not promote T cell activation and cell cycle progression. (A and B) Resting CD4 T cells were treated with N9 (10 μM) or a scrambled peptide (N20, 10 μM). As a control, cells were also activated with PHA (2 μg/ml) + IL-2 (2 ng/ml). Expression of CD25 (A) or CD69 (B) was quantified with labeled anti-CD25 or anti-CD69 antibodies and flow cytometry. (C) Actinator N9 does not promote cell cycle progression. Resting CD4 T cells were treated with N9 (10 μM) or a scrambled peptide (N20, 10 μM). As a control, cells were also activated with PHA (2 μg/ml) + IL-2 (2 ng/ml) and then analyzed with flow cytometry for cell cycle progression using 7-AAD, PY staining.

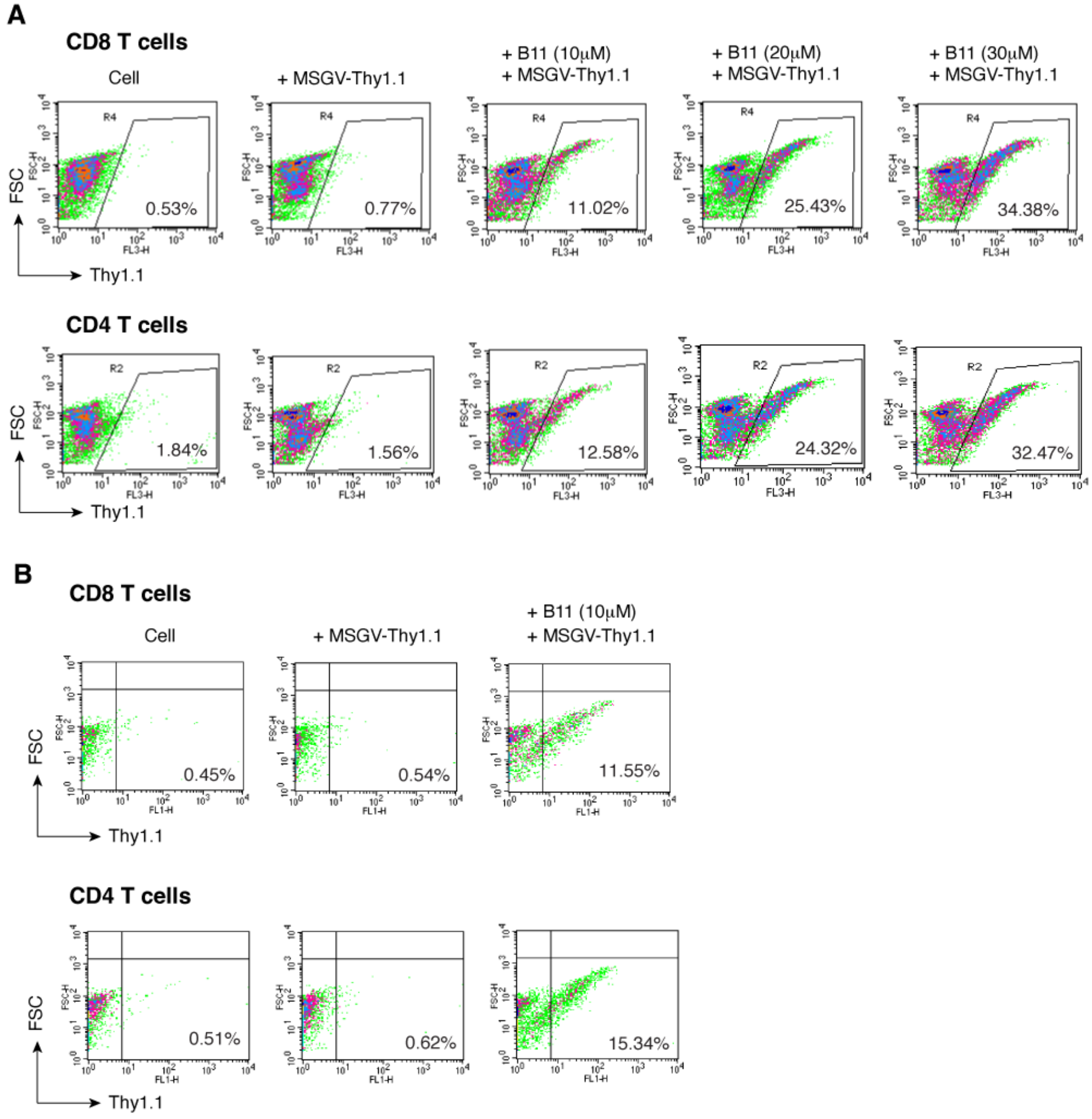

Fig. S8.

Actinator B11 enhances retroviral vector transduction of mouse CD4 and CD8 T cells. Mouse T cells were purified, and then activated with anti-CD3/CD28 antibodies. Cells were pre-treated or not with B11 for 1 hour and then infected with MSGV-Thy1.1 retroviral particles for 4 hours. Cells were washed, cultured, and then analyzed for Thy-1.1 expression with flow cytometry. (A) and (B) are two independent experiment repeats.

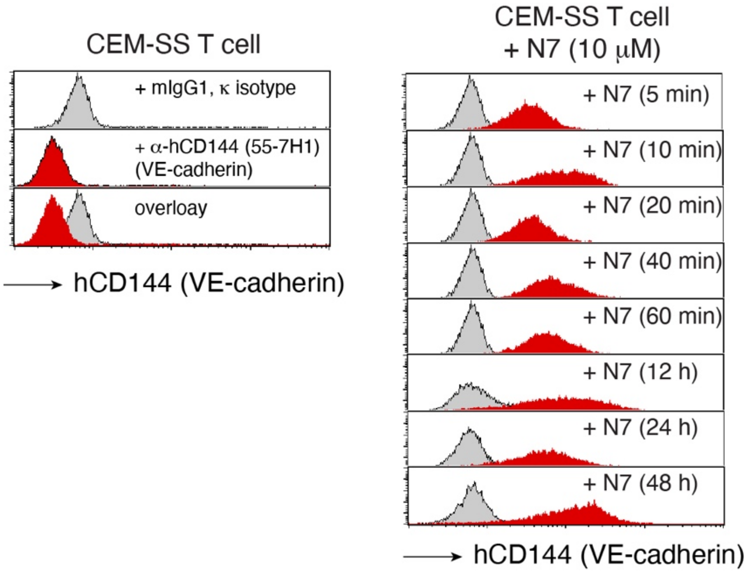

Fig. S9.

N7 enhances surface staining of CD144 (VE-cadherin) on CD4 T cells. CEM-SS T cells were treated with N7 for a time course, stained with a labelled anti-CD144 antibody, and then analyzed by flow cytometry.

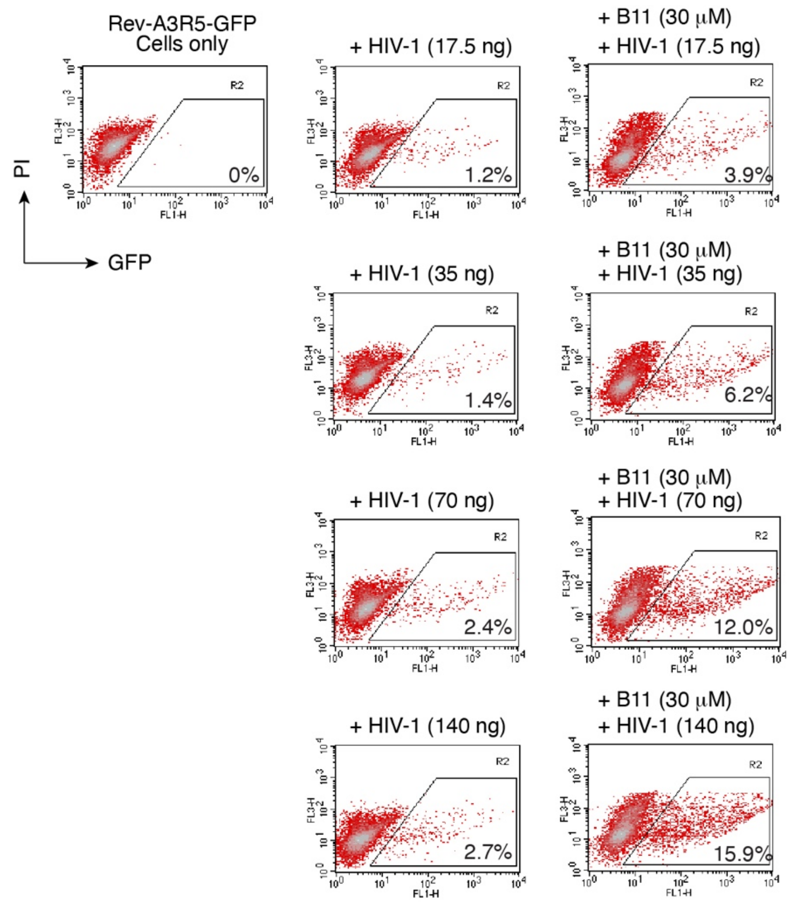

Fig. S10.

Actinator B11 enhances HIV-1 infection of Rev-A3R5-GFP T cells in various virus doses. Cells were pre-treated with B11 for 30 minutes and then infected with various doses of HIV-1 for 2 hours. Cells were washed and cultured for 2 days and then analyzed with flow cytometry for GFP expression. Propidium iodide (PI) was used during flow cytometry to quantify HIV-1 replication only in live cells.

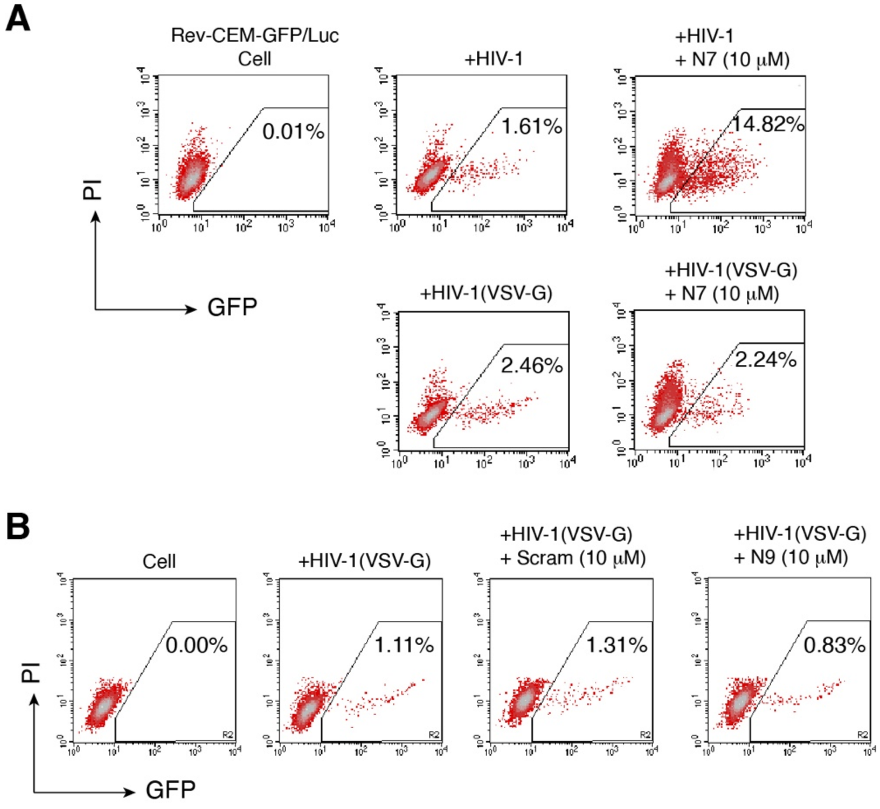

Fig. S11.

Differences between individual actinator peptides in enhancing HIV-1 versus VSV-G pseudotyped HIV infection. (A) HIV Rev-dependent GFP indicator cell, Rev-CEM-GFP/Luc, was pre-treated with N7 (10 μM) and then infected with HIV-1(NL4-3) or VSV-G pseudotyped HIV-1 for 2 hours, washed to remove the peptide and HIV-1, and then cultured for 2 days. HIV-1 replication was quantified by GFP expression. Propidium iodide (PI) was used during flow cytometry to quantify HIV-1 replication only in live cells. (B) Cells were also pre-treated with N9 (10 μM) and similarly infected with VSV-G pseudotyped HIV-1, and then analyzed.

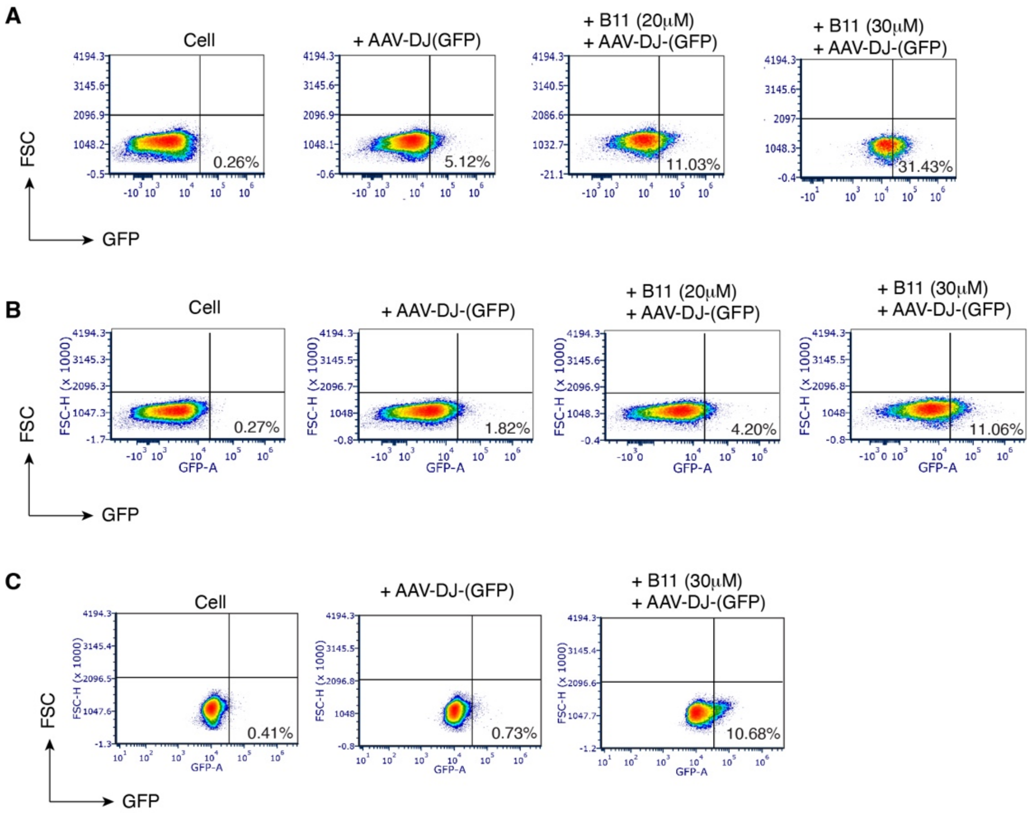

Fig. S12.

B11 enhances low-level AAV vector uptake into human T cells. Human CEM-SS T cells were pre-treated with B11 for 10 minutes and then transduced with AAV-DJ(GFP) viral particles for 2 hours. Cells were washed, cultured for 2 days, and then analyzed by flow cytometry for GFP expression. (A) to (C) are three independent experiment repeats.

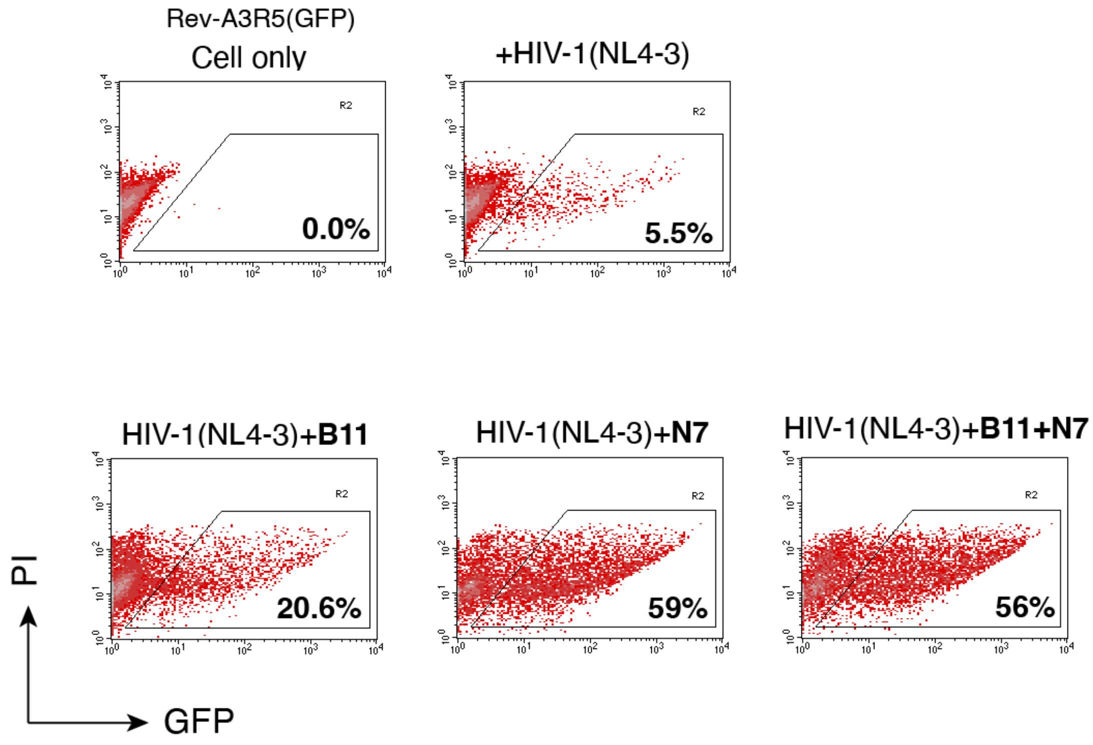

Fig. S13.

Effects of B11 plus N7 combination on enhancing HIV infection. Rev-A3R5-GFP indicator T cells were pre-treated with B11 (10 μM), N7 (10 μM), or B11+N7 (10 μM of each) for 30 minutes and then infected with HIV-1 for 2 hours. Cells were washed, cultured for 2 days, and then analyzed by flow cytometry for GFP expression.

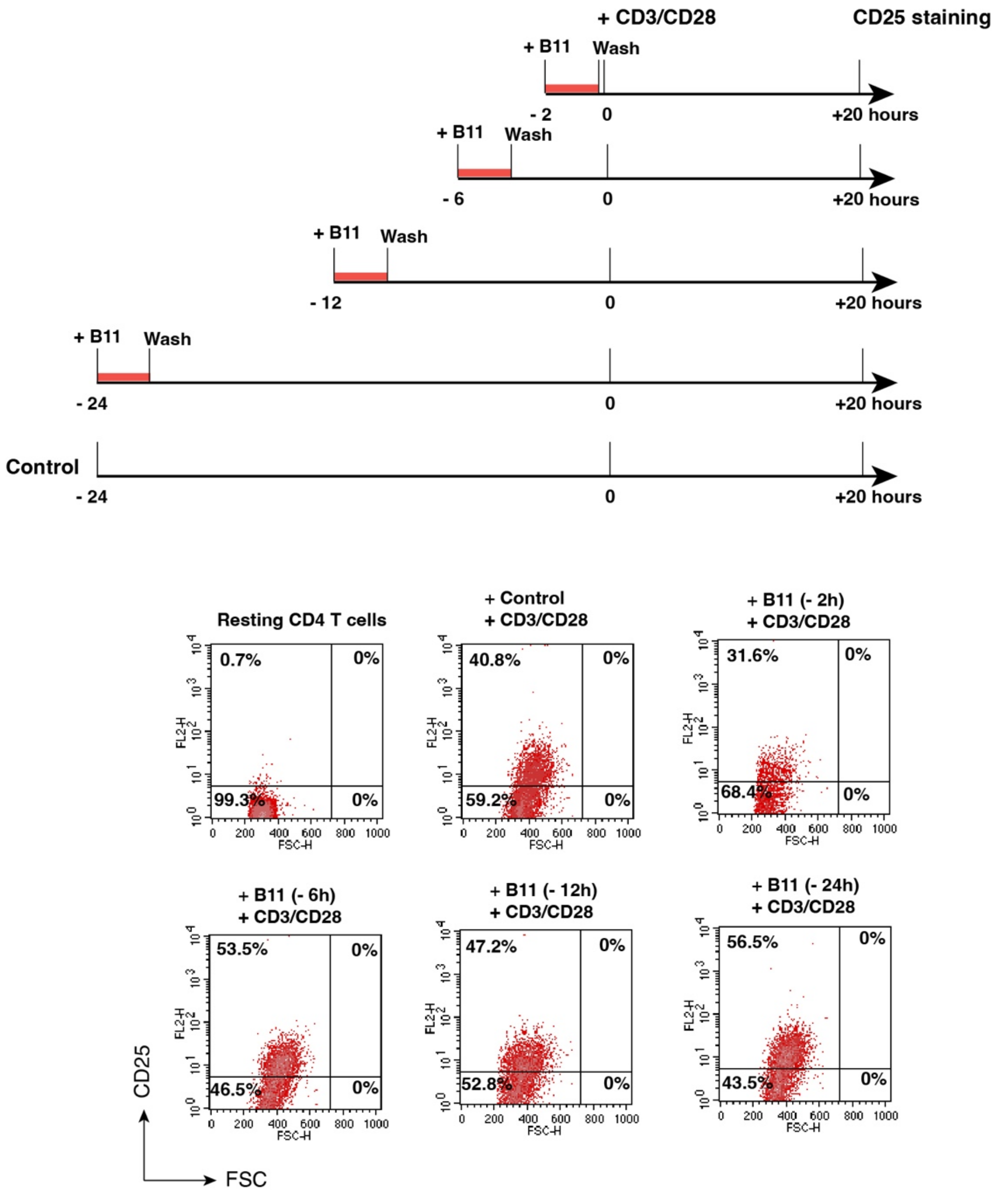

Fig. S14.

Effects of actinator B11 on CD4 T cell activation. Human CD4 T cells were purified from peripheral blood and cultured overnight. Cells were incubated with B11 (20 μM) for 2 hours, washed, and then activated (CD3/CD28 stimulation) immediately (-2h), or at 6 (-6h), 12 (-12h), or 24 (-24h) hours post treatment with B11. Activation of T cells was monitored by surface staining of CD25 upregulation. Un-activated resting CD4 T cells or T cells activated in the absence of B11 treatment were used as controls.

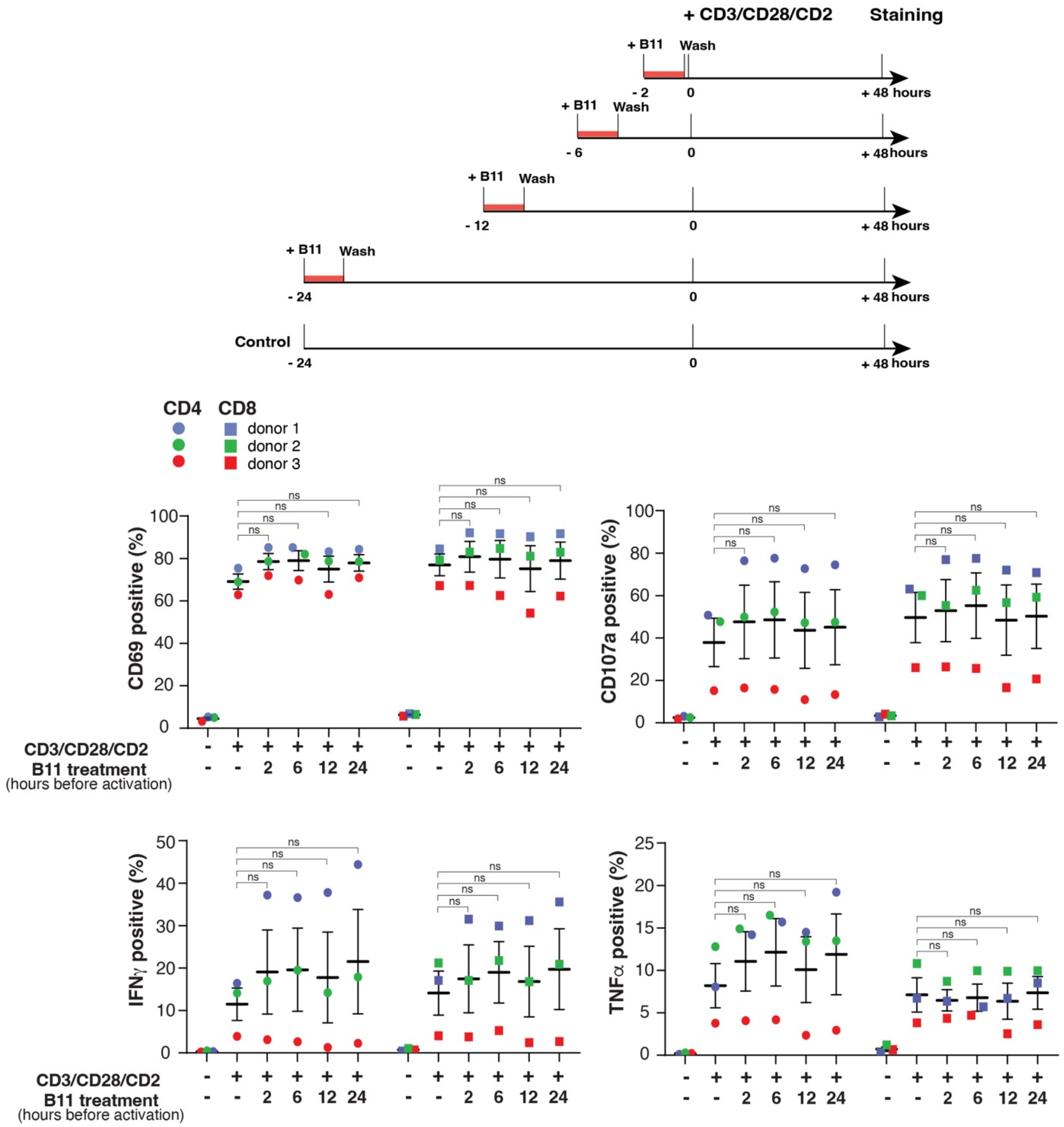

Fig. S15.

Effects of actinator B11 on CD4 (circle) and CD8 (square) T cell activation and functionality. Human T cells were purified from the PBMC of three healthy donors (colored blue, green, and red, respectively). Cells were incubated with B11 (20 μM) for 2 hours, washed, and then activated immediately, or at 6, 12, 24 hours post treatment with B11. T cells were activated by ImmunoCult (CD3/CD28/CD2) or un-activated. Activation of T cells was monitored by surface staining for CD69 upregulation. T cell functions were also measured by intracellular staining of CD107a, IFN-γ and TNF-α. The percentages of positively stained cells were quantified by flow cytometry, and the averages were plotted (black line). *P* values comparison for each time point was calculated using a parametric paired t-test, *ns*, not significant ($P > 0.05$).

Supplementary Table 1

Target	Fluorochrome	Vender	Clone	Cat#
hCD4	SB780	eBioscience	SK3	78-0047-42
hCD4	PE	eBioscience	PRA-T4	12-0049-42
mCD4	BV421	BD Biosciences	GK1.5	562891
hCD8	APC/Cy7	BioLegend	SK1	344714
mCD8	APC/Cy7	BioLegend	53-6.7	100714
hCD25	PE	BD Biosciences	BC96	567214
hCD69	PE	BD Biosciences	L78	341652
CD69	PE	BioLegend	FN50	310906
CD144	FITC	BD Biosciences	55-7H1	560411
CD324	PE	BD Biosciences	67A4	562870
CD325	RB670	BD Biosciences	8C11	770187
CXCR4	PE	BioLegend	12G5	306506
CD107a	BV650	BioLegend	H4A3	328638
IFN-γ	FITC	eBioscience	4S.B3	11-7319-82
TNF-α	PerCP/Cy5.5	BioLegend	MAb11	502926
mCD90.1(Thy1.1)	PE/Cy7	BioLegend	OX-7	202518
BODIPY 493/503		Thermo Fisher		D2191
mouse IgG1, κ	FITC	BioLegend	MOPC-21	400108
mouse IgG1, κ	PE	BioLegend	MOPC-21	400111
mouse IgG1, κ	PerCP/Cy5.5	BioLegend	MOPC-21	400147
mouse IgG2a, κ	PE	BioLegend	MOPC-173	400212
